# DELTEX E3 ligases ubiquitylate ADP-ribosyl modification on protein substrates

**DOI:** 10.1126/sciadv.add4253

**Published:** 2022-10-05

**Authors:** Kang Zhu, Marcin J. Suskiewicz, Andrea Hloušek-Kasun, Hervé Meudal, Andreja Mikoč, Vincent Aucagne, Dragana Ahel, Ivan Ahel

**Affiliations:** ^1^ Sir William Dunn School of Pathology, University of Oxford, Oxford, UK.; ^2^ Centre de Biophysique Moléculaire, CNRS UPR 4301, Orléans, France.; ^3^ Division of Molecular Biology, Ruđer Bošković Institute, Zagreb, Croatia.

## Abstract

Ubiquitylation had been considered limited to protein lysine residues, but other substrates have recently emerged. Here, we show that DELTEX E3 ligases specifically target the 3′ hydroxyl of the adenosine diphosphate (ADP)–ribosyl moiety that can be linked to a protein, thus generating a hybrid ADP-ribosyl-ubiquitin modification. Unlike other known hydroxyl-specific E3s, which proceed via a covalent E3~ubiqutin intermediate, DELTEX enzymes are RING E3s that stimulate a direct ubiquitin transfer from E2~ubiquitin onto a substrate. However, DELTEXes follow a previously unidentified paradigm for RING E3s, whereby the ligase not only forms a scaffold but also provides catalytic residues to activate the acceptor. Comparative analysis of known hydroxyl-ubiquitylating active sites points to the recurring use of a catalytic histidine residue, which, in DELTEX E3s, is potentiated by a glutamate in a catalytic triad-like manner. In addition, we determined the hydrolase specificity profile of this modification, identifying human and severe acute respiratory syndrome coronavirus 2 (SARS-CoV-2) enzymes that could reverse it in cells.

## Teaser

A noncanonical ubiquitylation reaction catalyzed by DTX enzymes generates a hybrid ADP-ribose-ubiquitin protein modification.

## INTRODUCTION

**Fig. 1.**
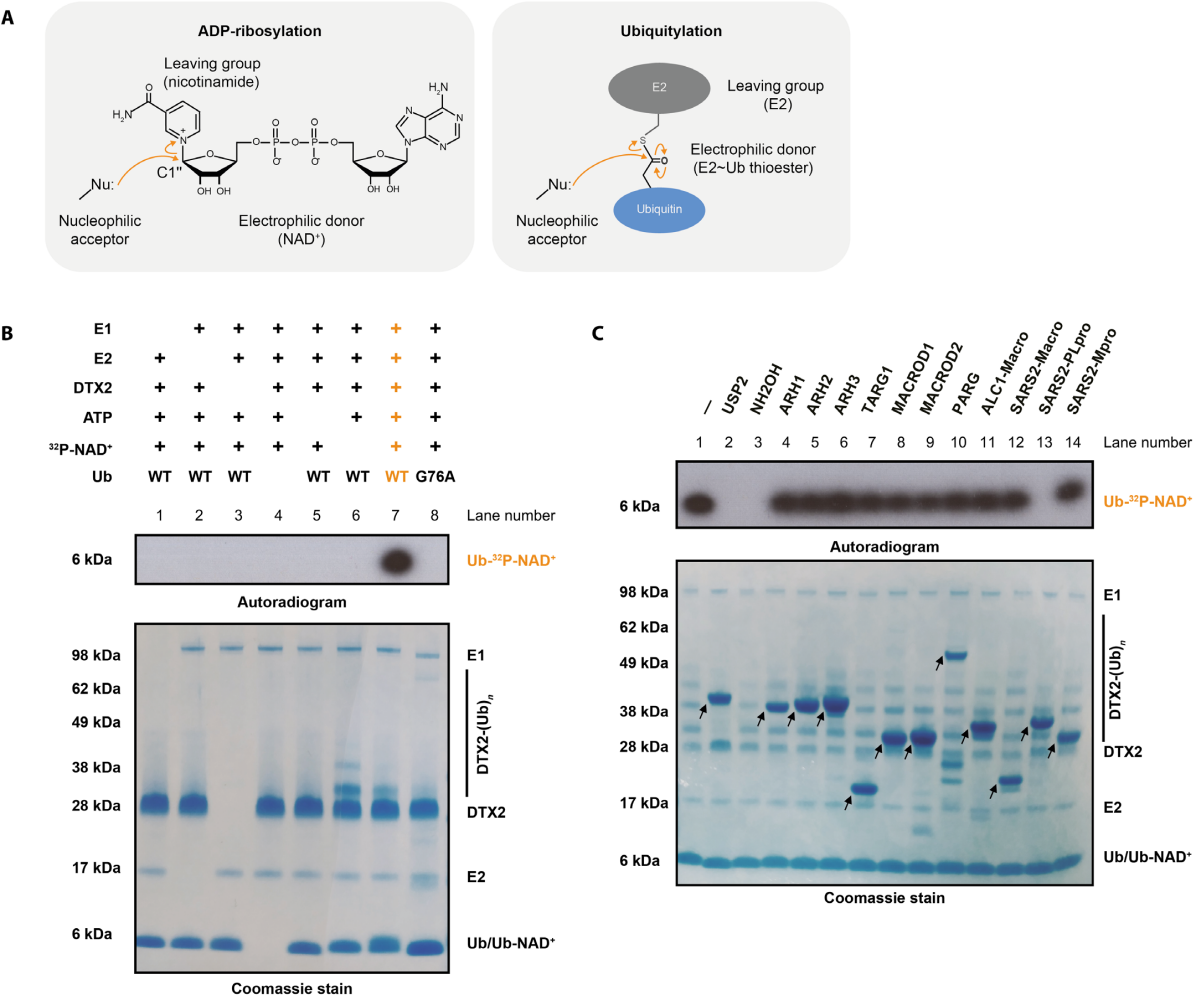
Biochemical determinants of Ub-NAD^+^ conjugate synthesis and hydrolysis. (**A**) Reaction mechanisms of ADP-ribosylation and ubiquitylation. Both modifications require a nucleophilic acceptor group. (**B**) Biochemical reconstitution of the DTX reaction. Ub-NAD^+^ is obtained by incubation of ^32^P-NAD^+^ with DTX2 RING-DTC (residues 390 to 622) and processed WT Ub, E1, E2, and ATP. The samples were analyzed on an SDS–polyacrylamide gel electrophoresis (SDS-PAGE) gel, which was then visualized by Coomassie staining (whole gel) and autoradiography (a fragment corresponding to Ub). Omitting any of these components or mutating Gly^76^ of Ub to Ala prevents conjugation. (**C**) Hydrolase sensitivity of Ub-NAD^+^. Following a reaction like in (B), the indicated ADPr hydrolases, DUBs or NH_2_OH, were added to the substrate and further incubated. The arrows indicate various hydrolases.

The attachment of the small protein modifier ubiquitin (Ub) to protein substrates, known as protein ubiquitylation or ubiquitination, is a central eukaryotic protein posttranslational modification (PTM) that controls protein activity, interactions, localization, and half-life (*1*, *2*). Ubiquitylation proceeds via a multienzyme cascade composed of a Ub-activating enzyme (E1), a Ub-conjugating enzyme (E2), and a Ub ligase (E3). Ub is first activated in an adenosine triphosphate (ATP)–dependent manner by an E1 and then transferred to the catalytic cysteine of an E2 to form the E2~Ub thioester (*3*). Subsequently, an E3 catalyzes the transfer of Ub to an acceptor moiety in a substrate (typically a protein lysine residue) (Fig. 1A), which can happen by one of two general mechanisms (*4*).

The most prevalent Ub ligase type, canonical really interesting new gene (RING) E3s, accelerate the direct handover of Ub from E2~Ub onto a substrate. To do so, these E3s bring the substrate and the E2~Ub conjugate together and stabilize, with their signature RING finger domain, the E2~Ub conjugate in the active “closed” conformation required for efficient lysine ubiquitylation (*5*–*7*). E3s that contain a U-box domain, which is related to the RING, operate in the same manner (*4*). In addition to this scaffold-like mechanism, a growing number of Ub ligases of various types, including HECT (homologous to the E6-AP carboxyl terminus) and RBR (RING-between-RING) classes, as well as MYC-binding protein 2 (MYCBP2) and RING finger protein 213 (RNF213), have been reported to work by accepting Ub from E2 onto a cysteine residue in their own sequence, thus forming a covalent E3~Ub thioester intermediate before depositing Ub onto a substrate (*4*, *8*–*10*).

Unlike canonical RING- and U-box–containing E3s, which in all known cases promote ubiquitylation of protein lysine residues, some of the E3s that proceed via the E3~Ub intermediate have been shown to modify hydroxyl acceptors. The ubiquitylated hydroxyl groups can be found either in proteins (especially threonine residues) (*10*, *11*) or, according to very recent data, in nonproteinaceous molecules such as lipopolysaccharide or glucosaccharide, which has opened new avenues of Ub research (*12*, *13*). In addition, one E2 enzyme, UBE2J2, has been reported to be capable of directly ubiquitylating hydroxyl groups in proteins without an E3 (*14*, *15*).

Ubiquitylation is in close cross-talk with other PTMs, including protein adenosine diphosphate (ADP)–ribosylation (*16*, *17*). ADP-ribosylation proceeds from nicotinamide adenine dinucleotide (NAD^+^) as a donor of the ADP-ribosyl (ADPr), which is attached to a protein substrate via the 1′ carbon of the adenine-distal ribose (hitherto called C1″), accompanied by simultaneous departure of the activating nicotinamide moiety (Fig. 1A) (*18*). The initial ADPr unit can be extended to a poly(ADPr) (PAR) chain in the process known as PARylation, mainly through linkages between the adenine-proximal 2′ hydroxyl of the preceding ADPr and C1″ of the succeeding one (*18*, *19*). One aspect of the cross-talk between ADP-ribosylation and ubiquitylation is represented by bacterial pathogens that use ADPr to manipulate Ub signaling in eukaryotic hosts. The effectors CteC from *Chromobacterium violaceum* and SidE from *Legionella pneumophila* attach a single ADPr to specific surface residues on the host Ub (Thr^66^ and Arg^42^, respectively), making Ub unusable by the host E1-E2-E3 cascade (*20*–*22*). In addition, SidE contains the phosphodiesterase activity that allows it to process Ub-attached ADPr to phosphoribose, which can either serve as the final inactivating modification or, in a proportion of molecules, be used to link Ub to serine residues in protein substrates in a noncanonical manner (*20*, *21*). An interplay between ADP-ribosylation and ubiquitylation is also observed in eukaryotes themselves, where PAR chains installed on a protein substrate by poly(ADP-ribose) polymerase (PARP) family ADP-ribosyltransferases can serve as an initial signal that is recognized by some Ub E3s, leading to ubiquitylation and, ultimately, degradation (*23*). Thus, the RING-containing PAR-targeted E3 RNF146 can recognize PAR via its WWE domain (named after its three conserved amino-acid residues), an interaction module named after three conserved amino acid residues, which allosterically activates the adjacent RING domain (*24*–*26*).

A potentially more complex but still unclear relationship between the two PTMs is mediated by the DELTEX (DTX) family of RING-containing Ub E3 ligases, which, in humans, comprises five enzymes (DTX1, DTX2, DTX3, DTX3L, and DTX4) (*27*, *28*), one of which, DTX3L, forms a stable complex with the putative ADP-ribosyltransferase PARP9 (*29*, *30*). All DTX E3s contain a signature DTX C-terminal domain (DTC) of a previously unclear function that is connected to the catalytic RING domain via a short flexible linker (*31*, *32*). In addition, DTX E3s have a long N-terminal extension that, in DTX1, DTX2, and DTX4, harbors tandem PAR-binding WWE domains. A recent study of DTX2 demonstrated its PAR-targeted ubiquitylation activity, which, unexpectedly, does not depend on its two WWE domains for PAR recognition, instead relying for this task on DTC (*31*). DTC can bind an ADP-ribose molecule, and, therefore, the RING-DTC fragment could potentially recruit a PARylated substrate to the E2~Ub thioester for ubiquitylation on lysine residues.

In addition to PAR-targeted protein ubiquitylation, DTX ligases were also shown to catalyze a reaction between NAD^+^ and E2~Ub (*32*). This unusual process was first described for the DTX3L:PARP9 complex (*33*) and subsequently shown to be catalyzed by an isolated RING-DTC fragment of DTX3L and the equivalent regions of other DTX ligases (*32*). The reaction was proposed to involve nicotinamide displacement from NAD^+^ and the attachment of ADPr via C1″, such as in canonical ADP-ribosylation, to the C-terminal carboxyl of Ub. This would yield C-terminally ADP-ribosylated, inactivated Ub as a product, possibly as a way of down-regulating Ub signaling (*33*). However, the proposed linkage was not directly demonstrated, and the functional relevance of the reported adduct remains unclear.

Here, we report that DTX-family Ub E3 ligases ubiquitylate ADP-ribosylated proteins and peptides on the ADPr modification in vitro, producing a potential PTM that combines ADPr and Ub in one covalent adduct. We identify the 3′ hydroxyl of the adenine-proximal ribose ring of ADPr as the ubiquitylation site, which means that Ub and a protein substrate can be attached at the opposite ends of a single bridging ADPr unit (Ub-ADPr-protein) or, potentially, of a PAR chain (Ub-[ADPr]*_n_*-protein). DTX E3s can also catalyze a reaction between Ub and NAD^+^ as previously described, but we detect Ub-NAD^+^ rather than ADP-ribosylated Ub as a product, consistent with the attachment happening on the 3′ hydroxyl and not C1″; moreover, NAD*^+^* ubiquitylation is disfavored relative to equivalent ADP-ribose modification and might represent a nonphysiological side reaction. The study identifies the first Ub E3 ligases that are capable of modifying a nonlysine acceptor via a direct transfer from the E2~Ub thioester, without an E3~Ub intermediate. This is possible because of a previously unidentified catalytic paradigm for RING-containing E3 ligases, whereby the E3 not only recruits the substrate to an activated E2~Ub but also uses specific catalytic residues to increase the nucleophilicity of the hydroxyl acceptor. In that regard, we identify a potential catalytic His-Glu pair that is conserved in the DTC domain of DTX E3s and forms a catalytic triad-like arrangement with the 3′ hydroxyl of ADPr to activate it for ubiquitylation. Moreover, our comparative analysis shows that the presence of a catalytic histidine is a recurring feature of all known hydroxyl-ubiquitylating E2 and E3 enzymes. Last, we determine the hydrolase sensitivity profile of the Ub-ADPr- modification, identifying enzymes that could remove it in cells in the context of an antiviral response or other pathways. Overall, these results suggest a notable previously unidentified example of noncanonical ubiquitylation that broadens the known spectrum of possible Ub chemistries.

## RESULTS

### The DTX reaction product can be cleaved by deubiquitylases including severe acute respiratory syndrome coronavirus 2 papain-like protease but not by ADPr hydrolases

The Paschal and Huang groups reported an enzymatic process that is catalyzed by DTX E3 ligases and involves NAD^+^ and Ub as substrates (*32*, *33*). Both groups proposed that the reaction results in ADP-ribosylated Ub as a product, but clear evidence of nicotinamide departure from NAD^+^ and formation of the ester bond between C1″ and the Ub C terminus has been lacking. We attempted to reproduce this process and conclusively identify its product. In agreement with previous reports, the RING-DTC fragment, which was identified by the Huang group as the minimal catalytic region (*32*), of DTX2 was capable of incorporating radioactivity from ^32^P-labeled NAD^+^ into Ub, indicating that either all of NAD^+^ or its part that includes the labeled moiety (adenosine-proximal phosphate) becomes coupled to Ub (Fig. 1B, lane 7). We refer to this radioactive adduct as the “DTX product.” Coomassie stain also revealed DTX2 autoubiquitylation (likely on lysine residues), which decreased upon NAD^+^ addition (compare lanes 6 and 7), suggesting that the two DTX-catalyzed processes—the reaction between NAD^+^ and Ub and DTX2 autoubiquitylation—are in competition with each other. As reported previously (*32*, *33*), the formation of the DTX product only took place in the presence of E1 and E2 enzymes (in our case, UBA1 and UBCH5A, respectively) and ATP and with wild-type (WT) but not G76A Ub, suggesting that NAD^+^ reacts with the E2~Ub thioester rather than free Ub (Fig. 1B, lane 7 contrasted with lanes 1 to 6 and 8). While expected on the basis of the mentioned recent studies, this struck us as paradoxical, because canonical ADP-ribosylation via the electrophilic C1″ atom requires a nucleophilic acceptor, but the E2~Ub thioester is itself an activated, electrophilic molecule that tends to react with nucleophiles (Fig. 1A). As suggested by Chatrin *et al.* (*32*), this conundrum could be solved if either NAD^+^ or E2~Ub becomes hydrolyzed before the final reaction. However, we also considered a simpler scenario where, contrary to what was proposed, no nicotinamide displacement takes place and, instead, Ub is attached to one of the existing nucleophilic moieties on NAD^+^, such as its hydroxyl groups.

To probe the chemical nature of the investigated product, we subjected it to hydrolysis by a panel of enzymes with different specificities. This set comprised several human hydrolases, including ADPr hydrolases that are specific for bonds between C1″ of ADPr and different acceptor groups, as well as the deubiquitylase (DUB) Ub-specific protease 2 (USP2), which cleaves various chemical bonds (including amide and ester) directly after Gly^76^ of Ub and has already been shown to cut the DTX-reaction product (*32*). As DTX E3s have been linked to immune responses against viruses (*34*, *35*), we also included three severe acute respiratory syndrome coronavirus 2 (SARS-CoV-2) enzymes: the Nsp3 macrodomain (Macro), which is an ADPr hydrolase; the main protease (Mpro), which has been reported to cleave a specific peptide sequence; and the papain-like protease (PLpro), which exhibits a DUB activity (*36*, *37*). The radioactive product was hydrolyzed by both tested DUBs—PLpro (but not its inactive mutant) and USP2—which was in line with the attachment happening via Gly^76^ but did not provide further clues about the chemical bond involved (Fig. 1C, lanes 2 and 13, and fig. S1A). We further confirmed that PLpro can cleave radioactive DTX-reaction products formed in reactions catalyzed by DTX2 and DTX3L RING-DTC fragments (fig. S1B). While the resistance of the product to Mpro (Fig. 1C, lane 14) and to the nonhydrolytic Macro domain of ALC1 (Fig. 1C, lane 11) was expected, unexpectedly, the analyzed product was also resistant to all tested ADPr hydrolases (lanes 4 to 10 and 12). Although it is not directly known which ADPr hydrolases could cleave ADPr off a terminal carboxyl moiety in a protein, the ester bond between C1″ and the C terminus would be chemically equivalent to glutamate- or aspartate-linked ADP-ribosylation, which can be reversed by MACROD1, TARG1 (*38*), and SARS-CoV-2 Macro (*39*, *40*). Notably, ADPr hydrolases generally do not require any specific sequence context beyond the hydrolyzed bond, and ADPr attached to the carboxyl of the flexible terminal Gly-Gly motif of Ub should be particularly accessible to enzymatic processing; both these considerations suggest that MACROD1, TARG1, and SARS-CoV-2 Macro should remove canonical ADP-ribosylation from Gly^76^ of Ub. Thus, resistance to these enzymes (Fig. 1C, lanes 8, 7, and 12) provided first hints that the DTX product might not correspond to ADP-ribosylated Ub.

In addition to testing enzymatic hydrolysis, we also treated the DTX product with hydroxylamine (NH_2_OH), which breaks ester and phosphoanhydride but not peptide or glycosidic bonds (Fig. 1C, lane 3). As observed before (*32*, *33*), the radioactive adduct was successfully hydrolyzed with this compound. NH_2_OH sensitivity would be consistent with ADP-ribosylation of the terminal Ub carboxyl (an ester bond), but it does not provide a conclusive proof, because it could also be explained by other scenarios, including ubiquitylation of NAD^+^ on one of its hydroxyl groups (also an ester bond). In addition, the interpretation is complicated by the fact that NH_2_OH would likely cleave the central phosphoanhydride bond within NAD^+^/ADPr in addition to possibly the linkage that connects the dinucleotide to Ub. Thus, while we were able to reproduce NH_2_OH sensitivity, our data together still argued against previously proposed identity of the DTX product.

Overall, these data provided the sensitivity profile of the identified adduct, suggesting that, in vivo, the bond between an NAD^+^ derivative and Ub could be regulated by human and viral DUBs, but not ADPr hydrolases or canonical proteases, and offered first indications that Ub might not be linked to C1″ of ADPr but instead possibly to one of the hydroxyls in NAD^+^.

### DTX E3s catalyze NAD^+^ and ADP-ribose ubiquitylation

We next attempted to track the product of the analyzed reaction using high-performance liquid chromatography–mass spectrometry (HPLC-MS) (fig. S2). In line with the above considerations, we did not detect a molecule that would correspond to the C-terminally ADP-ribosylated Ub (9106 Da). Instead, the predominant detected species had a mass of 9211 Da, which corresponds to the sum of NAD^+^ and Ub minus a water molecule (Table 1). This indicated that NAD^+^ still retains nicotinamide on C1″ and thus has to be attached to Ub via another atom, ruling out canonical ADP-ribosylation. We also performed the DTX reaction using an NAD^+^ analog, Carba-NAD^+^, which is inert in reactions that require displacement of nicotinamide from C1″ (fig. S3) (*41*). Carba-NAD^+^ could be efficiently ubiquitylated in a DTX-dependent manner (Table 1), reinforcing the idea that the reaction does not involve C1″. The reaction also proceeded with the C1″-hydrolyzed form of NAD^+^, i.e., free ADP-ribose, generating the Ub-ADPr adduct with an expected mass (Table 1). Overall, these results strongly suggested that DTX attaches Ub to NAD^+^ or ADP-ribose through an atom other than C1″.

**Table 1.** HPLC-MS identification of products of DTX2-catalyzed reactions performed with indicated substrates. Major detected masses and corresponding products are provided. All substrates except Carba-NAD^+^ were used at 4 mM. Carba-NAD^+^ was used at 100 μM because of its limited supply. Product identification and quantification was performed as illustrated in fig. S3. Chemical formulas of the analyzed substrates are available in fig. S2.

**Substrate**	**Detected mass of** **product (relative** **abundance)**	**Identified product,** **its average** **theoretical mass**
NAD^+^	9210.0 Da (92%)	Ub-NAD^+^, 9211.2 Da
	9104.6 Da (8%)	Ub-ADP-ribose, 9106.0 Da*
Carba-NAD^+^	9207.9 Da (85%)	Ub-Carba NAD^+^, 9209.2 Da
ADP-ribose	9104.7 Da (>90%)	Ub-ADP-ribose, 9106 Da
ADP	8972.6 Da (>90%)	Ub-ADP, 8973.9 Da
AMP	8892.7 Da (>90%)	Ub-AMP, 8893.9 Da
Adenosine	8812.6 Da (25%)	Ub-Adenosine, 8814.0 Da
	9052.7 Da (>50%)	Ub-ATP, 9053.9 Da
2′-deoxy ADP-ribose	9088.7 Da (>90%)	Ub-2′-deoxy ADP-ribose, 9090.0 Da

*Ub-ADP-ribose likely originates from ADP-ribose contamination present in the NAD^+^ stock due to its partial hydrolysis or from hydrolysis of Ub-NAD^+^ following the ubiquitylation reaction.

### DTX E3s conjugate Ub to the adenine-proximal part of NAD^+^ or ADP-ribose

**Fig. 2.**
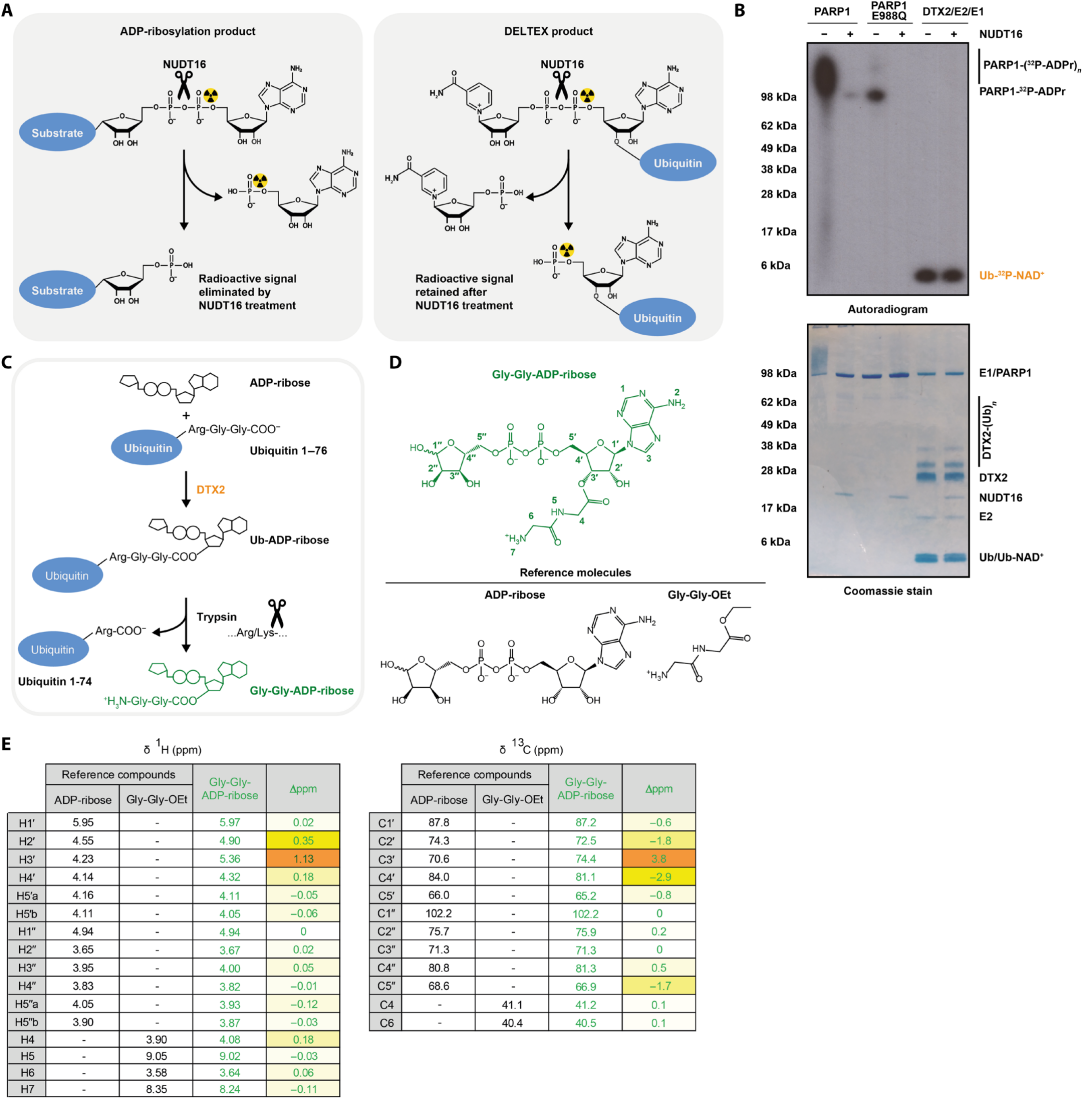
DTX E3s catalyze conjugation of Ub to the 3′ hydroxyl of NAD^+^/ADP-ribose. (**A**) Schematic diagram of expected NUDT16 cleavage specificity. NUDT16 treatment eliminates radioactivity from a ^32^P-NAD^+^–labeled protein if it is attached to the dinucleotide via C1″ but not when it is attached to the adenine-proximal ribose ring. (**B**) Poly(ADP-ribosyl)ated PARP1 WT, mono(ADP-ribosyl)ated PARP1 E988Q, and the DTX product were created by incubating the relevant components with NAD^+^ and then treated or not with NUDT16. The samples were analyzed on an SDS-PAGE gel, which is then visualized by Coomassie staining and autoradiography. NUDT16 reverses PARP1/PARP1 E988Q automodification but has no effect on the DTX2-catalyzed Ub-NAD^+^ adduct, consistent with the adenine-proximal Ub attachment. (**C**) Flowchart of Gly-Gly-ADP-ribose generation. The DTX reaction followed by trypsin digestion results in ADP-ribose attached to the tryptic Ub remnant Gly-Gly-. Trypsin cleavage specificity is indicated. (**D**) Chemical formulas of Gly-Gly-ADP-ribose and reference molecules used in NMR. (**E**) NMR localization of the Gly-Gly remnant to the 3′ hydroxyl group of the proximal ribose of ADP-ribose based on the largest shifts (Δppm) in δ ^1^H and ^13^C values in these positions. For ADP-ribose and Gly-Gly-ADP-ribose, δ ^1^H and ^13^C values are provided only for the major β anomer (full data and explanation in the Supplementary Materials).

Next, we attempted to narrow down the point of Ub attachment. NAD^+^ and ADP-ribose consist of two ribose rings that are joined by two phosphoryl moieties (i.e., a pyrophosphate) (fig. S3). The central phosphoanhydride bond can be cleaved enzymatically by diphosphatases including nucleoside diphosphate linked to moiety x (NUDIX) hydrolase 16 (NUDT16) (Fig. 2A) (*42*, *43*). As mentioned above, radioactive NAD^+^ is ^32^P-labeled on the adenine-proximal phosphoryl, so disappearance or persistence of the radioactivity in the adduct after NUDT16 treatment allows distinguishing which half of the dinucleotide serves at the attachment point. In a control experiment, NUDT16 could remove radioactive signal from radioactive NAD^+^-treated PARP1 WT (automodification with PAR chains) and PARP1 E988Q (automodification with a single ADPr unit), consistent with canonical ADP-ribosylation through C1″ on adenine-distal ribose (Fig. 2B). In contrast, the radioactive signal was retained in the DTX-catalyzed Ub-NAD^+^ adduct following NUDT16 treatment, suggesting that Ub is attached via the adenine-proximal half of NAD^+^. We performed a similar experiment with unlabeled Ub-ADP-ribose using MS to characterize the product after NUDT16 treatment. The determined mass corresponded to Ub–adenosine monophosphate (AMP), consistent with the initial Ub attachment being to the adenine-proximal half of the ADP-ribose molecule (the AMP fragment) (Table 2). Consistent with this result, DTX could ubiquitylate a free ADP and AMP molecule, producing Ub-ADP and Ub-AMP, respectively, as verified with MS (Table 1). To further narrow down the linkage, we attempted to use adenosine as a substrate, observing very inefficient but clear formation of Ub-adenosine. All these results are consistent with each other and clearly narrow down the point of attachment to the adenosine part of NAD^+^/ADP-ribose.

**Table 2.** HPLC-MS identification of products of cleavage reactions. Major detected masses and corresponding products are provided, with the dominant one for each reaction emphasized in bold. Product identification and quantification were performed as illustrated in fig. S3.

**Substrate and cause** **of cleavage**	**Detected mass of** **product (relative** **abundance)**	**Identified product,** **its average** **theoretical mass**
Ub-ADP-ribose cleaved with NUDT16	**8892.7 (85%)**	**Ub-AMP, 8893.9 Da**
	8637.6 (15%)	Ub-glycerol, 8638.8 Da*
Ub-ADP-ribose cleaved with NH_2_OH	**8578.7 Da (85%)**	**Ub-NHOH, 8579.8 Da**
	8594.2 (15%)	Ub-NHOH, +15 or 16 Da^†^

*Glycerol was present in the NUDT16 stock. We hypothesize that the DTX:E2 complex can catalyze Ub conjugation to hydroxyl groups in glycerol, similarly to what was reported for another hydroxyl-ubiquitylating enzyme, MYCBP2 (*10*).

^†^This mass difference could conceivably correspond to a reaction of NH_2_OH with Ub aspartimide (possibly formed on Asp^52^ of Ub).

### DTX E3s ubiquitylate the 3′ hydroxyl of NAD^+^ or ADP-ribose

Next, we considered different possible nucleophilic acceptors within the identified fragment. As discussed above, the Ub-NAD^+^ adduct can be chemically cleaved with NH_2_OH (*44*). We demonstrated that the same is the case for Ub-ADP-ribose and used MS to confirm that the cleavage with NH_2_OH results in Ub-NHOH (Table 2). Although we cannot rule out that Ub linked through an adenosine amide, which is expected to be slightly more reactive to nucleophiles than a peptide or isopeptide amide, would be fully resistant to NH_2_OH, efficient cleavage with NH_2_OH is known to be a hallmark of ester-linked ubiquitylation, making it the most likely hypothesis in the light of current knowledge. Therefore, we hypothesized that Ub attachment might occur at the 2′ or 3′ hydroxyl moieties on the adenine-proximal ribose. DTX-catalyzed reaction performed using 2′-deoxy ADP-ribose generated the Ub-dinucleotide adduct, speaking against the first of these options (Table 1). While we could not test in parallel 3′-deoxy ADP-ribose because of unavailability of this analog, our result points to 3′ hydroxyl as a possible candidate.

To more directly probe the connection between Ub and ADP-ribose, we used partial trypsin treatment to process Ub-ADP-ribose to Gly-Gly-ADP-ribose (Fig. 2C and fig. S4) (*45*), which was subsequently HPLC-purified and subjected to nuclear magnetic resonance (NMR) analysis. Release of Gly-Gly-ADP-ribose with trypsin, in addition to DUB sensitivity of the analyzed adducts, provides unambiguous proof of the attachment being to the C terminus of Ub rather than (as would theoretically be consistent with some of our observations, e.g., NH_2_OH sensitivity) an Asp or Glu residue on Ub. During the HPLC step, we were unable to separate Gly-Gly-ADP-ribose from free AMP (produced by the E1 enzyme during the ubiquitylation reaction), resulting in a mixture containing around 15% Gly-Gly-ADP-ribose and 85% AMP (fig. S5). We confirmed the presence of both species with MS and analyzed the sample with NMR. The obtained spectra are fully consistent with a weighted sum of the spectra for AMP and ADP-ribose with the Ub remnant attached to its 3′ hydroxyl moiety. Briefly, acquisition of two-dimensional (2D) ^1^H total correlation spectroscopy (TOCSY) and ^1^H-^13^C heteronuclear single-quantum coherence (HSQC) spectra allowed us to unambiguously identify most proton and CH/CH_2_ carbon signals of the two compounds, and chemical shifts were compared to those of commercial compounds used as references: AMP, ADP-ribose, and Gly-Gly-ethyl ester (see the Supplementary Materials for the spectra and details) (Fig. 2D and figs. S6 and S8). As shown in Fig. 2E and table S1, a strong deshielding [>1.1 parts per million (ppm)] of the H3′ proton of the proximal ribose was observed, as classically seen upon the formation of an ester derivative of an alcohol. As expected, H2′ and H4′ were also deshielded (0.35 and 0.18 ppm, respectively), and most other protons were barely affected. Consistently, C3′ carbon was shifted downfield, while neighboring C2′ and C4′ signals moved upfield (Fig. 2E and table S2). These balanced effects on chemical shifts of carbon atoms in the α and β positions relative to the 3′ oxygen are fully consistent with expectations (table S3). Together, our biochemical and NMR analyses consistently identify 3′ hydroxyl of NAD^+^ and ADP-ribose as the main or possibly the only point of Ub attachment, identifying the DTX reaction as a previously unidentified example of hydroxyl ubiquitylation.

### Molecular mechanism of NAD^+^/ADP-ribose ubiquitylation by DTX E3s

**Fig. 3.**
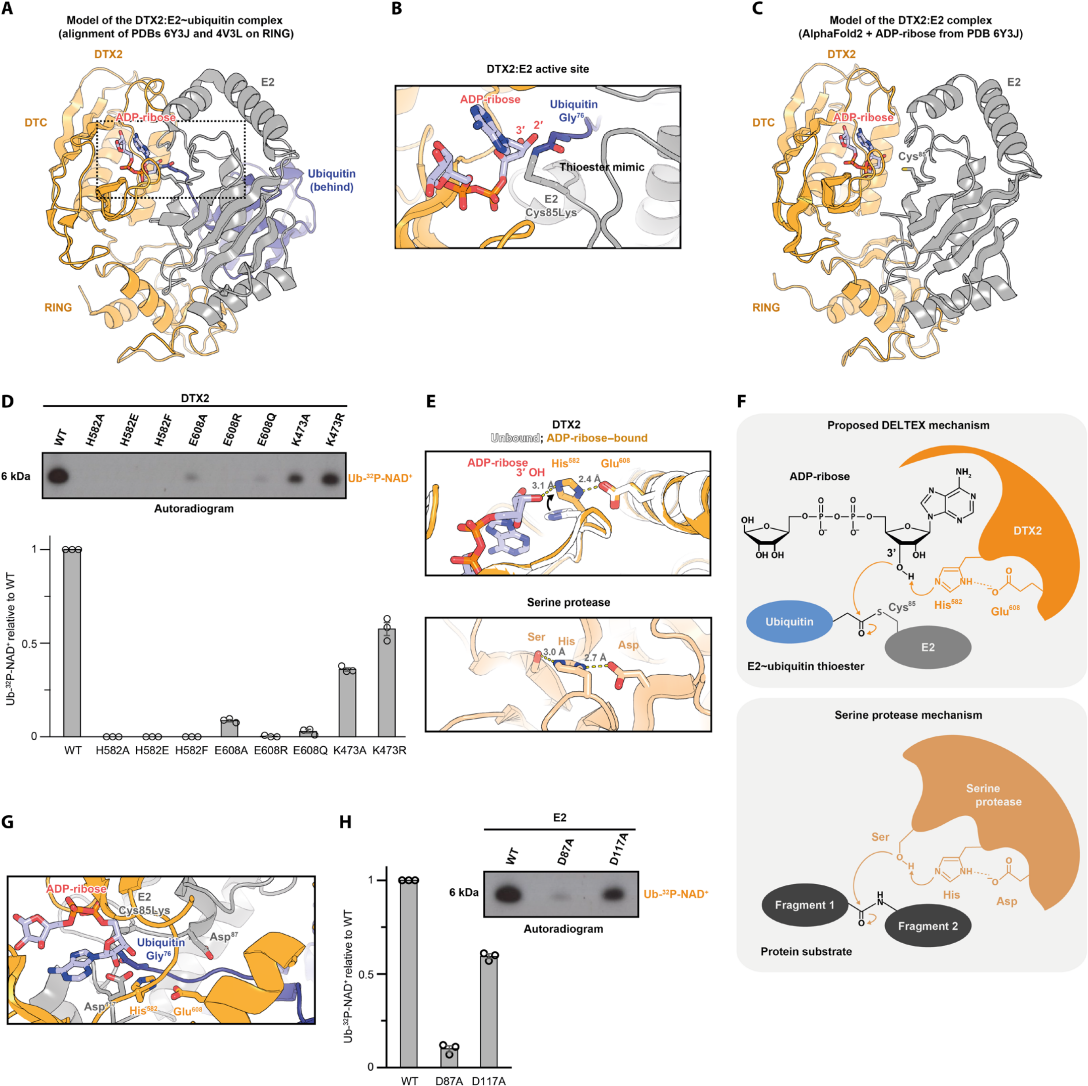
Mechanism of NAD^+^/ADP-ribose ubiquitylation by DTX E3s. (**A**) A model of a complex between an E2~Ub mimic, DTX2 RING-DTC, and ADP-ribose obtained by aligning, on the RING domain, DTX2 RING-DTC:ADP-ribose [Protein Data Bank (PDB): 6Y3J] with RNF38:UBCH5A~Ub (PDB: 4V3L). RNF38 is not shown. The fragment zoomed in (B) is indicated. (**B**) The active site of the complex from (A). The Cys85Lys mutation produces a stable isopeptide bond between E2 and Ub that mimics a thioester. (**C**) An AlphaFold2 model of a complex between DTX RING-DTC and the E2 UBCH5A. ADPr is inserted on the basis of the PDB 6Y3J. (**D**) Mutational analysis of DTX2 using the NAD^+^ ubiquitylation assay introduced in Fig. 1B. The product was visualized with autoradiography and quantified. The bar graph shows the mean ± SEM for *n* = 3 independent assays of the same protein preparations. Full results are in fig. S10A. (**E**) Comparison of the key residues involved in ADP-ribose ubiquitylation and the catalytic triad of a serine protease (α-chymotrypsin; PDB: 5CHA). His^582^ flips upon ADPr binding ~90° upward (comparison of PDBs 6Y22 and 6Y3J). (**F**) Reaction mechanisms of ADPr ubiquitylation by DTX2 and that of serine protease–mediated peptide cleavage. (**G**) Substrate lysine access to Asp^117^ is potentially sterically hindered by ADPr. (**H**) Mutational analysis of E2 in the presence of DTX2 RING-DTC using the NAD^+^ ubiquitylation assay introduced in Fig. 1B but with SUMO-E2 (WT or D117A) instead of E2. The results are quantified and presented as in (D), and full results are shown in fig. S10B.

The Huang group has obtained crystal structures of the RING-DTC fragments from DTX1 and DTX2 in ligand-free and ligand-bound states (NAD^+^ bound for DTX2 and ADP-ribose bound for DTX1) (*31*, *32*). In these structures, RING and DTC are connected by a linker that allows some flexibility of the two domains with respect to each other and likely accounts for their varying relative orientation in different crystal forms. Following demonstration of a direct interaction between the RING-DTC fragment and a stable E2~Ub mimic, the Huang group performed rigid-body structural alignments to model the complex between the two molecules (*31*, *32*). The DTX RING-DTC structures were aligned, on the RING domain, with a previously determined structure of a RING domain of another E3 ligase (RNF38) bound to a stable E2~Ub mimic (*46*). These analyses, which we have reproduced and extended, show proximity between the NAD^+^/ADP-ribose–binding site on DTC and the thioester bond in E2~Ub, with the two being either directly adjacent (Fig. 3A) or up to around 12 Å apart depending on the RING-DTC linker conformation (*32*). In line with our experimental data, the part of the DTC-bound ADP-ribose ligand that is closest to the E2~Ub thioester bond in the model is the adenine-proximal ribose ring that contains the 3′ hydroxyl (Fig. 3B). In contrast, the previously postulated attachment of ADPr through C1″, which is located on the opposite end of NAD^+^/ADP-ribose, would require DTC to substantially rotate relative to RING in a way that is not observed in any of the available structures and might not be feasible, despite linker flexibility. We also generated an AlphaFold prediction of the DTX2 RING-DTC:E2 complex, which is consistent with the models obtained from alignment (Fig. 3C).

We focused on the alignment obtained with the ADP-ribose–bound RING-DTC fragment of DTX2, in which DTC is closest to the E2~Ub thioester mimic (Fig. 3, A and B). In this model, there are some steric clashes between DTC and E2 (fig. S7A), but they involve two extended loops in DTC and could be alleviated if the loops altered their conformation upon E2 binding. Notably, the 3′ hydroxyl moiety of ADP-ribose, which we identified as the main acceptor of Ub in the DTX reaction, is located just 3.1 Å away from the C terminus of Ub, indicating that the state captured in this superimposition might be close to the arrangement required during the reaction. In this conformation, DTC and the ADP-ribose ligand sterically block the access to the thioester and adjacent catalytic E2 residues, which would provide a way of limiting lysine modification even if a given ADP-ribose:Ub encounter were to be unproductive (Fig. 3G).

Subsequently, we focused on the DTC domain and the way in which it interacts with the NAD^+^/ADP-ribose ligand (Fig. 3, B and E, and fig. S7B). By comparing the ligand-free and ADP-ribose–bound states of DTX2 RING-DTC characterized by the Huang group, we observed a movement of the His^582^ side chain, which flips down ~90° to coordinate hydroxyl moieties of the adenine-proximal part of ADP-ribose (Fig. 3E). The Huang group has previously determined the importance of this residue for ligand binding (*32*). In the ADP-ribose–bound state, His^582^ becomes inserted between the 3′ hydroxyl of ADP-ribose and Glu^608^ of the DTC domain, leading to a linear catalytic triad-like arrangement of hydroxyl, histidyl, and carboxyl moieties within hydrogen bonding distance from each other (Fig. 3, E and F). Considering that a hydroxyl is a relatively weak nucleophile and it might require activation to undergo ubiquitylation, we hypothesized that the observed arrangement might serve a catalytic role, deprotonating and thus activating the 3′ hydroxyl for the nucleophilic attack on the E2~Ub thioester. Consistent with an important role for His^582^ and Glu^608^, they are both strictly conserved among DTX proteins (fig. S9). We tested several substitutions in either of these residues in an NAD^+^ ubiquitylation reaction, finding that all these mutations impaired Ub-NAD^+^ formation (Fig. 3D and fig. S10A). Particularly notable is the marked effect of the conservative E608Q mutation, despite Glu^608^ not being in direct contact with ADP-ribose.

Next, we turned our attention to the DTX RING domain. A RING typically recruits the E2~Ub conjugate and stabilizes it in the closed conformation, which is a prerequisite for efficient protein lysine ubiquitylation (*5*–*7*). A key residue for this mechanism is the so-called “linchpin” arginine, which is situated directly after the last CXXC motif of the RING and interacts with both the E2 and Ub to constrain them with respect to each other (fig. S7C) (*7*). The linchpin arginine appears particularly important for activating canonical E2s from the UBCH5 family, for which the free E2~Ub conjugate rarely samples the closed conformation (*3*, *47*). However, as observed before by the Huang group (*32*), human DTX E3s, despite working with UBCH5 E2s, do not have an arginine in the linchpin position and instead have a lysine (DTX1, DTX2, and DTX4; see fig. S7C), glutamine (DTX3L), or even glycine (DTX3). According to a recent study focused on the linchpin’s role in Ub transfer, these residues are suboptimal for stabilizing the closed conformation of E2~Ub (*47*). Thus, although DTX E3s can become automodified on lysine residues in the presence of the E2~Ub thioester, pointing to their ability to exert a stabilizing effect on the closed conformation of E2~Ub, it is possible that these ligases evolved to do so inefficiently and thus avoid too strong stimulation of lysine ubiquitylation. This could allow them to instead favor an NAD^+^/ADP-ribose reaction, provided that it does not depend strongly on the closed E2~Ub state. To test the importance of the DTX RING linchpin for NAD^+^/ADP-ribose ubiquitylation, we mutated this residue in DTX2, Lys^473^, to either alanine or arginine, which should result in a RING that is either further impaired (K473A) or actually improved (K473R) in terms of stabilizing the closed E2~Ub conformation. Either of these mutations had a limited negative effect on Ub-NAD^+^ generation, although K473R was more efficient than K473A (Fig. 3D and fig. S10A). This suggests that NAD^+^/ADP-ribose ubiquitylation does not depend too strongly on the stabilized closed conformation of the E2~Ub thioester, perhaps because of efficient activation of the hydroxyl acceptor by His^582^ and Glu^608^ of the DTC domain proposed above.

Last, we investigated the importance of potential catalytic residues within the E2. During lysine ubiquitylation, the closed E2~Ub conformation positions Ub for the nucleophilic attack by the incoming lysine, which then interacts with the “gateway” aspartate conserved in some E2s. In the UBCH5 family E2s, this residue (Asp^117^ in UBCH5A) increases the nucleophilicity of the lysine acceptor, and its mutation severely compromises lysine-targeted activity (Fig. 3G and fig. S11 and S7B) (*48*–*50*). On the other hand, Asp^117^ is completely dispensable for hydrolysis of the E2~Ub ester (obtained by mutating the catalytic cysteine of E2, Cys^85^, to a serine), in which water serves as a nucleophile instead of lysine, or for transfer of Ub from the E2~Ub thioester onto a cysteine (*6*, *49*). This indicates that the function of Asp^117^ is lysine specific and does not extend to hydroxyl or thiol acceptors. That is not the case for another active-site aspartate residue in E2, Asp^87^, which is required for both lysine modification and E2~Ub ester hydrolysis, pointing to a more general role that might go beyond lysine modification. With these considerations in mind, we speculated that Asp^117^ but not Asp^87^ of E2 might be dispensable for NAD^+^/ADP-ribose ubiquitylation. While the D87A mutant of E2 was very inefficient at catalyzing either lysine-targeted DTX automodification or NAD^+^ ubiquitylation, the D117A mutant was only strongly compromised in the canonical lysine activity, while its hydroxyl-modifying potential remained high (Fig. 3H and fig. S10B). This establishes the E2 D117A as a separation-of-function mutant that blocks lysine modification but preserves DTX-catalyzed hydroxyl ubiquitylation.

Overall, the above structural alignments and biochemical data point to several differences between canonical RING-catalyzed lysine ubiquitylation and DTX-catalyzed NAD^+^/ADP-ribose ubiquitylation. The latter process requires two potential catalytic residues within the DTX DTC domain but only weakly relies on the stabilization of the closed conformation by the DTX RING domain and does not involve the gateway aspartate Asp^117^ of E2. These differences are consistent with the unprecedented chemistry of the DTX reaction, whereby Ub is transferred onto a hydroxyl acceptor directly from a UBCH5 E2~Ub conjugate.

### DTX E3s show a preference for ADP-ribose over NAD^+^

**Fig. 4.**
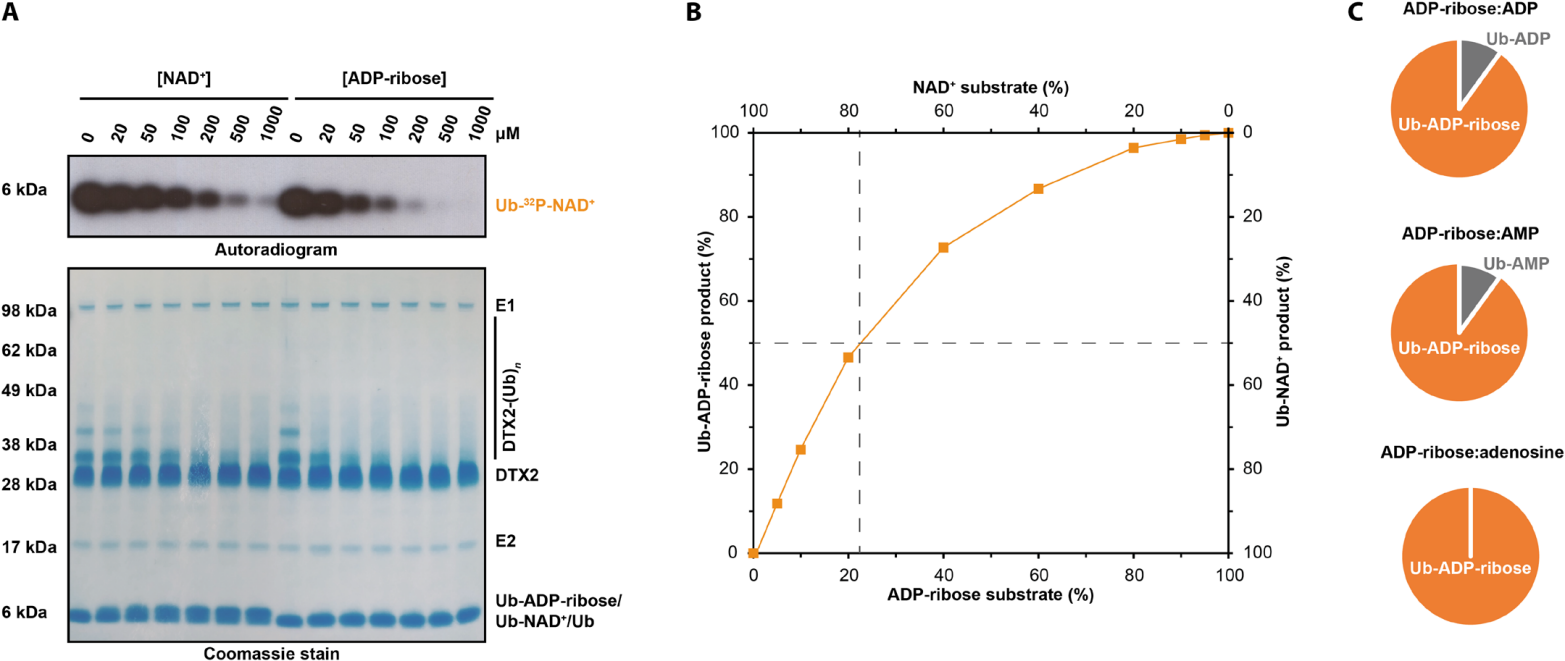
DTX E3 ligases show a preference for ADP-ribose over NAD^+^ and mononucleotides. (**A**) ADP-ribose shows stronger inhibition of DTX2 activity than NAD^+^ does, consistent with stronger binding. Indicated amounts of unlabeled NAD^+^ and ADP-ribose were titrated into a DTX2 reaction mixture, and their inhibitory effects on DTX2 activities were monitored using ^32^P-NAD^+^ (Ub-NAD^+^ formation) and on SDS-PAGE (DTX2 autoubiquitylation). (**B**) ADP-ribose is preferred over NAD^+^ as a substrate of DTX-catalyzed ubiquitylation. Different ratios of the ADP-ribose and NAD^+^ substrates (as indicated on the *x* axis) were incubated with DTX2 RING-DTC, E1, E2, Ub, and ATP, and the resultant products were identified and quantified with HPLC-MS as described in the Supplementary Materials. The accuracy of this analytical method has been estimated to be within the range of ±10%, assuming equal specific intensities for various small molecules being compared and linear dependence of intensity on concentration. The proportion of Ub-ADP-ribose and Ub-NAD^+^ products is shown on the *y* axis. Dashed lines indicate that for equimolar amounts of both substrates, the Ub-ADP-ribose product is estimated to be about four times more abundant than Ub-NAD^+^. (**C**) ADP-ribose is preferred over ADP, AMP, and adenosine as a substrate of DTX-catalyzed ubiquitylation. Equimolar amounts of ADP-ribose and indicated mononucleotides were used as substrates in a reaction mixture containing DTX RING-DTC, E1, E2, Ub, and ATP and the products analyzed with HPLC-MS as described in the Supplementary Materials. In each case, Ub-ADP-ribose constitutes at least ~90% of the products.

Considering that DTX E3s can ubiquitylate various substrates in vitro, we wondered which of them is preferred in vitro and thus potentially in cells. We performed a reaction with a low amount of ^32^P-labeled NAD^+^, adding increasing molarities of either cold NAD^+^ or cold ADP-ribose as a competitor. Both molecules inhibited the formation of the radioactive Ub-NAD^+^ adduct, but ADP-ribose did it approximately fivefold more efficiently than NAD^+^. The accompanying Coomassie-stained gel showed that titrating in either NAD^+^ or ADP-ribose also inhibited DTX RING-DTC automodification on lysine residues, and ADP-ribose again was a stronger competitor (Fig. 4A). To validate the observed preference for ADP-ribose over NAD^+^, we performed an MS-based analysis with nonradioactive NAD^+^ and ADP-ribose mixed in different proportions as a substrate of the DTX reaction. This experiment showed that equimolar mixture of Ub-ADP-ribose and Ub-NAD^+^ products could be obtained when using 20% ADP-ribose:80% NAD^+^ substrate mixture, in line with an approximately fourfold preference for ADP-ribose over NAD^+^ (Fig. 4B).

Above, we identified ADP, AMP, and adenosine as additional in vitro substrates of DTX-catalyzed ubiquitylation. To compare each of these substrates with ADP-ribose, we analyzed products obtained using 50% ADP:50% ADP-ribose, 50% AMP:50% ADP-ribose, or 50% adenosine:50% ADP-ribose mixtures. In each case, Ub-ADP-ribose constituted at least 90% of the products. Ub-ADP accounted for 10% of the products of the first mixture, and Ub-AMP also 10% of the second, while no Ub-adenosine could be detected (Fig. 4C). This suggests that ADP and AMP are around 10-fold less-efficient substrates than ADP-ribose, while adenosine is even more disfavored. The decrease in the efficiency of DTX catalysis for ADP, AMP, and adenosine, which are therefore unlikely to be physiological substrates, can most likely be explained by decreased binding of these fragments to the DTC, as crystal structures of NAD^+^- and ADP-ribose–bound RING-DTC demonstrate a contribution of the adenine-distal ribose ring and the pyrophosphate moiety to DTC binding.

Overall, these experiments point to the preference of DTX2 for ADP-ribose over NAD^+^ and other substrates, likely due to differences in affinity of these various ligands for the DTX DTC domain. However, the relative levels of ubiquitylation of NAD^+^, ADP-ribose, ADP, AMP, or any other possible substrates (including ADP-ribosylated proteins as shown below) would depend not only on binding preference but also on relative local concentrations of these potential substrates. Thus, the question of which substrates are most relevant in cells requires further study.

### DTX E3s ubiquitylate ADP-ribosylated peptides and proteins

**Fig. 5.**
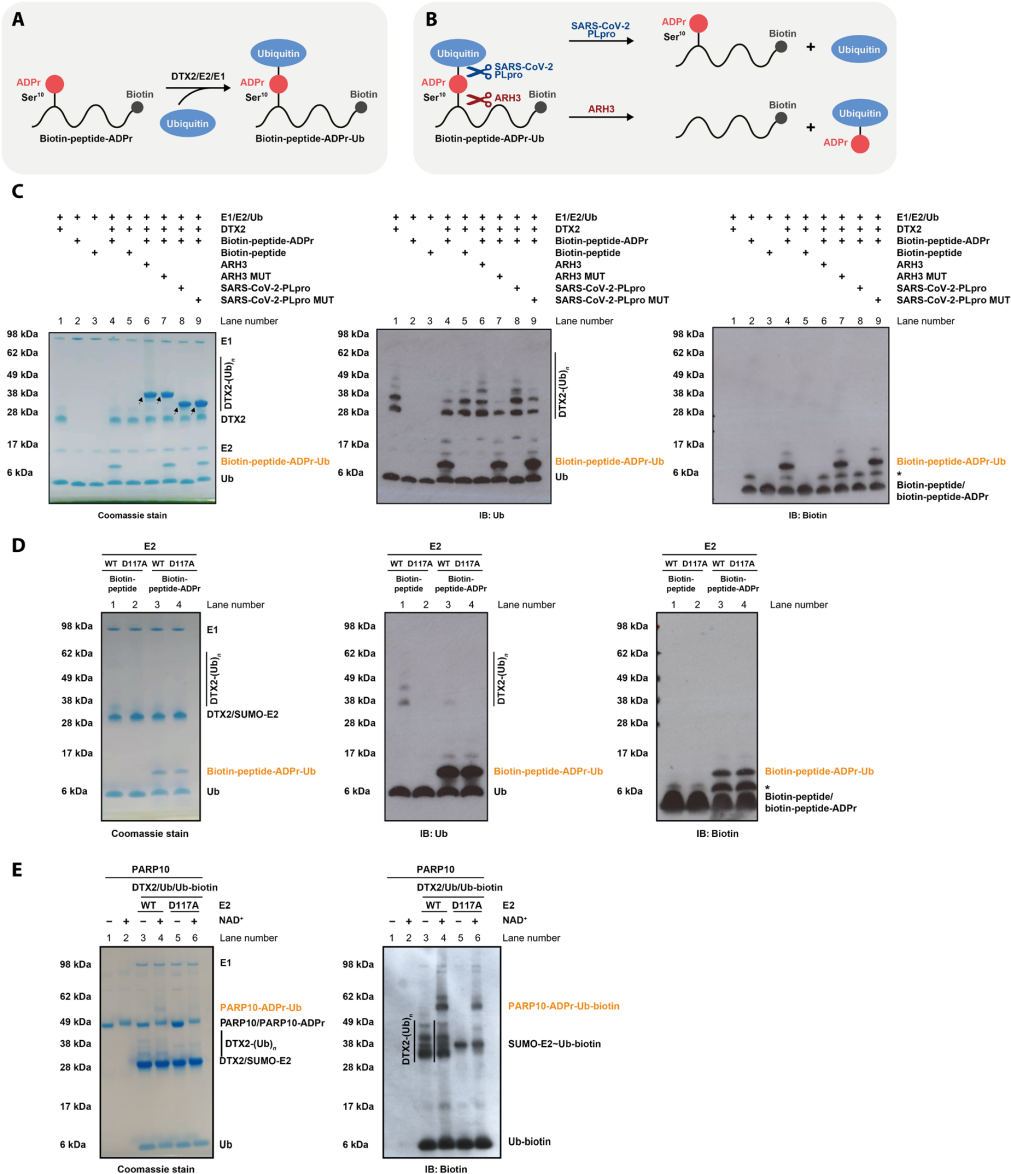
DTX E3 ligases ubiquitylate an ADP-ribosylated peptide and protein. (**A**) DTX2-catalyzed ubiquitylation of an ADP-ribosylated histone H3–derived peptide results in a peptide carrying a composite ADPr-Ub modification. (**B**) A schematic showing the cleavage sites of ARH3 and SARS-CoV-2 PLpro within peptide-ADPr-Ub. (**C**) The conjugate between Ub and an ADP-ribosylated peptide is obtained by incubation of histone H3–derived biotinylated peptide-ADPr with DTX2 RING-DTC (residues 390 to 622), Ub, E1, E2, and ATP. The same reaction with an unmodified biotinylated H3 peptide was performed as a control. The products were then incubated with indicated hydrolases, revealing sensitivity of the peptide-ADPr-Ub adduct to ARH3, consistent with ubiquitylation of peptide-ADPr on the ADPr moiety as illustrated in (B). The samples were analyzed by SDS-PAGE, and either the gel was stained with Commassie (left) or proteins were transferred onto a membrane and immunoblotted (IB) with anti-Ub (middle) or anti-biotin (right; detecting biotinylated peptides) antibodies. The arrows indicate ARH3 and PLpro, while the asterisk represents a contaminant present in peptide-ADPr. (**D**) An assay analogous to that in (C) probing the dependence of peptide-ADPr-Ub formation on Asp^117^ of E2. DTX2-catalyzed ubiquitylation of an ADP-ribosylated peptide in the presence of E1, E2, and ATP does not require Asp^117^ of the E2 UBCH5A, consistent with the ubiquitylation of the ADPr moiety. (**E**) PARP10 was preincubated with NAD^+^ or buffer and ubiquitylated with a mixture of unlabeled and biotinylated Ub by DTX2 RING-DTC (residues 390 to 622) in the presence of E1, E2, and ATP. Note that SUMO-tagged E2 was used. The results were visualized with a Coomassie stain (left) and anti-biotin antibody (right; detecting biotin-Ub). DTX2 ubiquitylates auto(ADP-ribosyl)ated PARP10 but not its unmodified form. The dispensability of Asp^117^ of E2 for the reaction suggests Ub attachment via ADPr.

DTX E3s have been described to have two different activities: protein ubiquitylation, which was reported to preferentially target ADP-ribosylated protein substrates, and the reaction between NAD^+^ and Ub, which we characterized above, extending it to free ADP-ribose as a substrate (*31*–*33*). We therefore wondered whether DTX E3s might combine these two functions by ubiquitylating ADP-ribosylated proteins indirectly by attaching Ub to the protein-linked ADPr modification. Because DTXs attach Ub to the adenine-proximal ribose of NAD^+^/ADP-ribose, it is conceivable that they work not only on free ADP-ribose but also on the ADPr moiety that was attached to a protein via the distal ribose through a canonical PARP-catalyzed ADP-ribosylation reaction. To explore this question, we first used a biotinylated histone H3–derived peptide modified on a serine residue with a single ADPr moiety [prepared as described in (*51*, *52*)] (Fig. 5A). This peptide was treated with the active DTX2 fragment in the presence of the Ub cascade components. To distinguish between ADPr-targeted peptide ubiquitylation on one of its lysine residues and ubiquitylation of the ADPr modification itself, we tested the sensitivity of the product to hydrolysis by ARH3, which is specific for the Ser-ADPr bond (Fig. 5B) (*53*). The product of the DTX reaction was monitored through a Coomassie-stained gel, which revealed a significant upward shift of Ub consistent with a covalent fusion between the ADP-ribosylated peptide and Ub and was confirmed by immunoblotting with anti-biotin and anti-Ub antibodies (Fig. 5C, lane 4). An equivalent product was not produced when a control peptide devoid of the ADPr modification was used (lane 5), indicating that the modification is ADPr dependent. WT ARH3, but not its inactive mutant, efficiently cleaved the peptide-ADPr-Ub product, as manifested in the loss of the upward shift (lanes 6 and 7, respectively), confirming that Ub is attached via the ADPr moiety (Ub-ADPr-peptide) presumably on its 3′ hydroxyl as in the case of free ADP-ribose or NAD^+^. This result also shows that the hydrolase ARH3 can cleave the bond between peptide and ADPr while the latter molecule is attached to Ub, which is consistent with structural data that show that ARH3 binds ADP-ribose in an orientation in which adenosine-proximal hydroxyl groups are facing the solvent (*54*). In addition, we tested SARS-CoV-2 PLpro, a DUB that was shown above to cleave the Ub-NAD^+^ adduct (Fig. 1C, lane 13). Its WT, but not inactive mutant, version digested the peptide-ADPr-Ub product (Fig. 5C, lanes 8 and 9, respectively). This means that the composite Ub-ADPr-peptide modification could be reversed in cells both by DUBs (on the level of the Ub-ADPr bond) and specific ADPr hydrolases (on the level of the ADPr-peptide/protein bond).

In a follow-up experiment, we modified peptide-ADPr-Ub using either WT E2 or the separation of function D117A mutant of E2, which abolishes lysine modification but above was shown to be largely dispensable for NAD^+^ ubiquitylation (Fig. 3H). Consistent with Ub being ligated to the ADPr modification, the reaction proceeded with similar efficiency with both WT and D117A E2 (Fig. 5D, lanes 3 and 4).

To extend this analysis to ADP-ribosylated proteins, we used DTX2 to ubiquitylate the catalytic domain of PARP10 (PARP10CAT) that was preincubated with NAD^+^ (which results in ADP-ribosylation of acidic residues in PARP10CAT with single ADPr units) (*55*, *56*) or remained unmodified. Coomassie staining and accompanying immunoblotting revealed PARP10 ubiquitylation that largely depends on PARP10CAT being auto(ADP-ribosyl)ated (Fig. 5E, compare lanes 3 and 4). To distinguish between ADPr-targeted lysine ubiquitylation and actual ADPr ubiquitylation, we again resorted to the separation of function D117A mutant of E2. While the D117A substitution precluded DTX2 autoubiquitylation, it had little effect on the ADPr-dependent PARP10CAT modification (Fig. 5E, compare lanes 4 and 6), indicating that ubiquitylation can be attached to the ADPr modification that is covalently linked to amino acid residues on a protein substrate. Overall, these experiments show that DTX E3s can promote ubiquitylation of model ADP-ribosylated peptides and proteins on the ADPr modification in vitro.

## DISCUSSION

DTX family E3 ligases have recently been shown to catalyze both PAR-targeted protein ubiquitylation and a reaction between NAD^+^ and E2~Ub of unclear nature and relevance (*29*, *32*, *33*). The latter process was proposed to lead to a bond between C1″ of ADPr and the C-terminal carboxyl moiety of Ub, possibly as a way of inactivating Ub. Contrary to this suggestion, our biochemical, MS, and NMR analyses demonstrate that DTX2 transfers Ub onto the 3′ hydroxyl in the adenine-proximal fragment of NAD^+^, ADP-ribose, or ADP-ribosylated proteins. In the last case, the reaction produces a hybrid Ub-ADPr- modification on a protein, which might have a distinct role in cellular signaling.

From a mechanistic perspective, the DTX-catalyzed hydroxyl ubiquitylation represents a previously unidentified catalytic paradigm that extends the spectrum of currently known ubiquitylation mechanisms. Following ATP-dependent Ub activation, the ubiquitylation cascade involves a series of Ub transfer events, initially between reactive cysteine residues and eventually from a cysteine in an E2 or an E3 to an acceptor group in a substrate (*57*, *58*). Currently, two types of final Ub acceptors are known: a lysine amino group or a hydroxyl moiety, where the latter can be part of a nonproteinaceous substrate (*12*, *13*). In all cases reported so far, substrate specificity is determined by the last enzyme that is covalently linked to Ub and creates a favorable local environment for subsequent transfer onto an appropriate acceptor. Thus, it has been assumed that, in instances where the substrate accepts Ub from a cysteine in an E2, it is always the E2 that defines the chemical spectrum of possible targets, and an E3 can only bias a predetermined choice by stabilizing a particular E2~Ub conformation. On the other hand, in cases where an E3 accepts Ub onto a cysteine in its own sequence, it is the E3 that determines the final substrate specificity and that of E2 becomes irrelevant. In line with this paradigm, the canonical E2s from the UBCH5 family, which have inherent cysteine- and lysine-ubiquitylating activity, cannot catalyze hydroxyl modification when associated with standard RING/U-box–containing E3s, which work by stimulating a direct transfer from E2 onto a substrate. However, MYCBP2, an atypical RING E3 that contains a reactive cysteine and forms a covalent E3~Ub intermediate, can bypass the inherent limitations of UBCH5 E2s and catalyze hydroxyl modification (*10*, *48*). Similarly, the E2 UBCH7, which is inherently active only toward cysteines, can participate in a cascade that leads to lysine or even hydroxyl ubiquitylation, as long as a suitable E3 bypasses a direct E2-to-substrate transfer of Ub by forming an E3~Ub intermediate (*8*, *12*).

Shedding unexpected light on these considerations, our study demonstrates, for the first time, a scenario where a RING-containing E3 enables a direct transfer of Ub from E2 to an acceptor that the same E2 would not be able to modify on its own. In this case, the role of the RING E3 goes beyond “chaperoning” a specific E2~Ub conformation and involves provision of catalytic residues for acceptor activation. The DTX E3s, and specifically their DTC domains, appear to perform the main catalytic role in the ADPr ubiquitylation reaction, while the catalytic Asp^117^ of the E2, which would be needed if a lysine was being modified (*6*, *49*), becomes largely dispensable. Moreover, the closed E2~Ub conformation seemingly does not need to be perfectly stabilized, as reflected in the suboptimal linchpin residues present in DTX RING domains, which likely makes them inefficient at this task (*47*). The catalytic determinants of the Ub transfer from UBCH5A onto the DTC-activated ADPr hydroxyl appear similar to those reported for the transfer from E2~Ub onto a cysteine catalyzed by the noncanonical RING E3 MYCBP2 (as a first step toward ultimate hydroxyl ubiquitylation) (*48*). This leads to the notion that the ADPr hydroxyl that is bound by the DTX DTC domain becomes similarly reactive to a thiol moiety.

What is the basis of this activation? By analyzing the DTX RING-DTC:ADPr crystal structure obtained by the Huang group (*31*, *32*), we identified a potential linear hydrogen-bonding cascade that consists of the 3′ ADPr hydroxyl and two highly conserved residues in the DTC domain, His^582^ and Glu^608^ (DTX2 numbering) (Fig. 3E). Although only the histidine is in direct contact with ADPr (and was previously shown to be important for ADPr binding), mutations in either residue, including the conservative E608Q substitution, have a marked effect on hydroxyl ubiquitylation, suggesting their catalytic roles. Moreover, the identified arrangement is notably similar to the catalytic triad of serine proteases, which is also composed of linearly arranged hydroxyl, histidyl, and carboxyl moieties, and is known to have evolved independently multiple times as a way of activating a serine residue for a nucleophilic attack on a peptide bond (Fig. 3F) (*59*). In the course of the multistep proteolysis reaction, the catalytic serine of these proteases forms a covalent ester intermediate with one part of the cleaved peptide, which is chemically analogous to a hydroxyl-Ub conjugate produced in the DTX reaction. DTC-catalyzed ADPr activation might therefore represent one more example of evolution stumbling upon the same solution for catalyzing peptidyl ester formation.

**Fig. 6.**
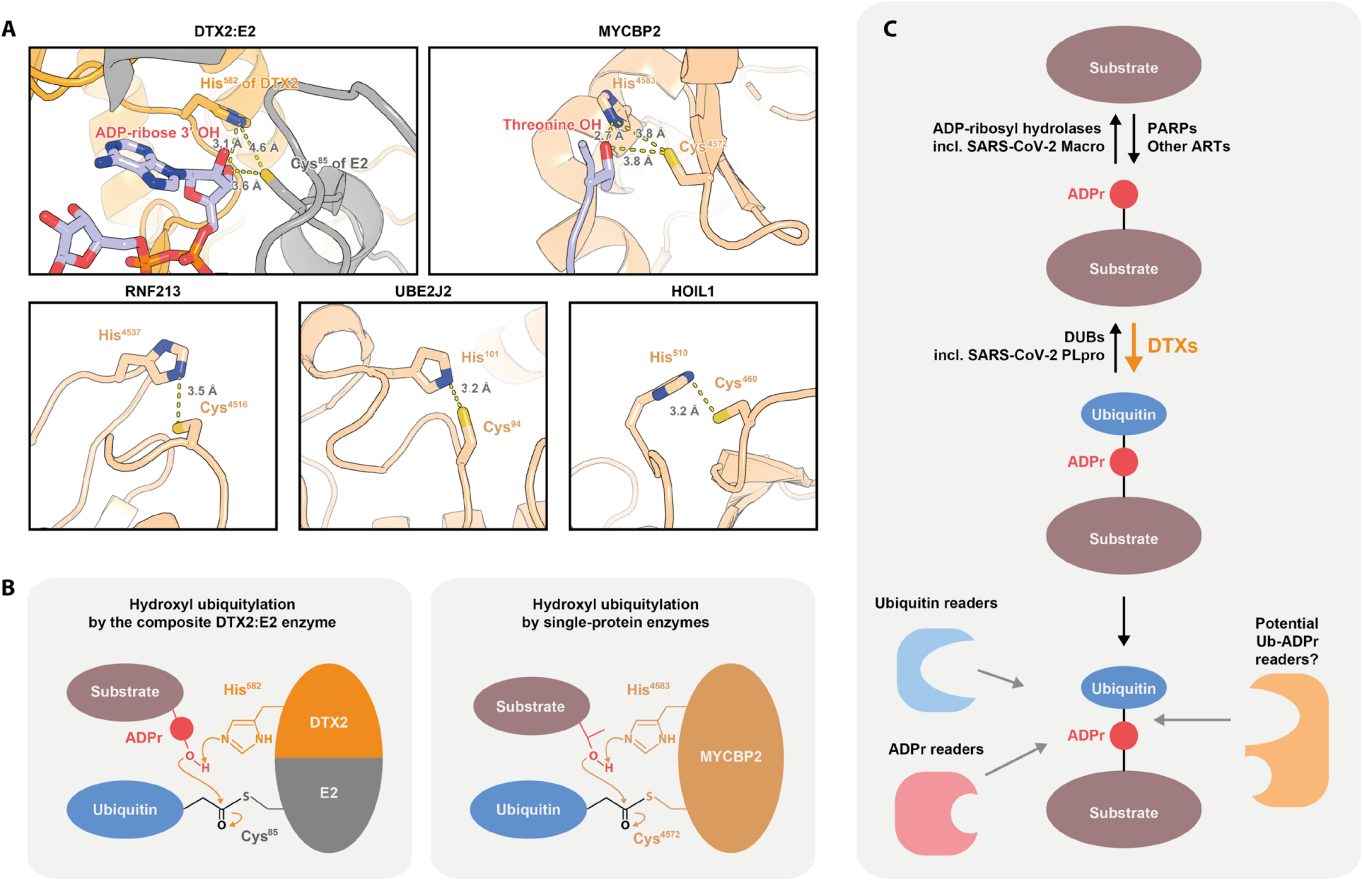
The molecular mechanism of hydroxyl group ubiquitylation and the possible model of regulation of and by the composite ADPr-Ub modification. (**A**) All known human hydroxyl-ubiquitylating E3 or E2 enzymes contain a putative catalytic His in spatial proximity to a Cys from which Ub is transferred onto a hydroxyl. For the DTX2:E2 composite enzyme, a fragment of a model produced by alignment of two structures (PDBs: 6Y3J and 4V3L) on the RING domain as introduced in Fig. 3A is shown. This model contains the ADP-ribose substrate molecule. For MYCBP2, a fragment of PDB 5O6C is shown, with a Thr residue from a crystallographic neighbor mimicking a hydroxyl substrate. For RNF213, UBE2J2, and HOIL1, AlphaFold2 models are shown. For HOIL1, the position of the His^510^ side chain (human numbering) is presented according to the AlphaFold2 models of murine and zebrafish HOIL1, which are consistent with each other (in the AlphaFold2 model of the human HOIL1, the His^510^ side chain is rotated away from Cys^460^, but the residue is still close to Cys^460^). (**B**) The proposed mechanism of hydroxyl ubiquitylation by the composite DTX2:E2 complex or MYCBP2. In both cases, a catalytic His might function as a general base that deprotonates the hydroxyl group undergoing modification (present within an ADPr modification or a Thr residue, respectively). (**C**) A putative pathway for synthesis, removal, and recognition of the composite ADPr-Ub modification. PARPs or other ADPr transferases (ARTs) provide ADP-ribosylated substrates that can then be ubiquitylated by DTX E3s. These reactions are sequentially reversed by DUBs (including SARS-CoV-2 PLpro) and ADPr hydrolases (including SARS-CoV-2 Macro). The composite ADPr-Ub modification could be recognized both by Ub and ADPr readers and potential specific Ub-ADPr–binding domains.

Conserved essential histidine residues have also been observed in the active sites of hydroxyl-modifying E3s that operate via an E3~Ub intermediate: RNF213 (His^4483^ in mouse, equivalent to 4537 in human) and MYCBP2 (His^4583^), suggesting that at least the catalytic histidine might be a relatively general feature of hydroxyl-ubiquitylating enzymes (Fig. 6A) (*8*, *10*). We looked for suitably positioned histidine residues in two other enzymes that have been reported to catalyze hydroxyl ubiquitylation but in which catalytic residues are unknown: the E2 UBE2J2 (*14*, *15*), which can do so in an E3-independent manner, and the E3 HOIL-1 (*11*, *12*). Analysis of AlphaFold models of these two proteins reveals the presence of suitable candidate residues, His^101^ of UBE2J2 and His^510^ of HOIL-1 (human numbering), both of which are highly conserved in orthologs on the primary structure level. In RNF213, MYCBP2, UBE2J2, and HOIL-1, the putative catalytic histidine residue is located on the same polypeptide as the cysteine from which Ub is then transferred onto the hydroxyl (Fig. 6A) (*10*, *13*). The unique feature of DTX-catalyzed ADPr ubiquitylation is that the catalytic histidine is provided by one protein (the DTX E3), while Ub is transferred from a cysteine in another (the E2), the two residues being brought into spatial proximity during the reaction (Fig. 6B). This is conceptually reminiscent of the composite active site built up by two proteins, HPF1 and PARP1, for catalyzing serine ADP-ribosylation (*60*). Previously, the Huang group reported that, in some, RING E3–specific non-RING elements contribute to ubiquitylation by activating donor Ub (*61*). Here, in a somewhat analogous fashion, DTX E3s provide a non-RING element (the DTC domain) for activating the other side of the reaction—the acceptor moiety. It is conceivable that “substrate-activating domain” analogs to DTX DTC evolved also for other substrates, allowing RING E3s to stimulate the direct transfer of Ub onto acceptor moieties that are not modified by isolated E2 enzymes.

As we show above, DTX E3s are able to ubiquitylate the 3’ hydroxyl of the adenosine-proximal part of the ADPr moiety, which is consistent with simultaneous attachment of ADPr through the C1″ atom to a protein substrate or another ADPr unit (in a PAR chain) (*18*, *19*). We directly demonstrate DTX-catalyzed ubiquitylation of ADP-ribosylated peptides and proteins in vitro. In addition to the mechanistic novelty discussed above, the reaction produces a potential previously unknown PTM signal, whereby Ub is linked to a substrate via a bridging ADPr unit. The Ub-ADPr- adduct not only combines two moieties that could be individually recognized by cognate reader domains but also represents a unique “hybrid chain” that could potentially recruit distinct readers that might be specific for the composite adduct and not respond to either ADPr or Ub in isolation from each other (Fig. 6C). By characterizing molecular determinants of the DTX reaction and the hydrolytic sensitivity profile of its product, we provide a stepping stone for the future functional analysis of this intriguing adduct in cells.

## MATERIALS AND METHODS

### Materials

We used the following commercially available materials: Ub antibody P4D1 (Insight Biotechnology, catalog no. sc-8017), streptavidin horseradish peroxidase (HRP) (Abcam, catalog no. ab7403), *Escherichia coli* Rosetta (DE3) competent cells (Novagen Merck, catalog no. 0954-3CN), HiLoad 16/600 Superdex 200 pg (Sigma-Aldrich, catalog no. GE28-9893-35), benzonase nuclease (Millipore-Merck, catalog no. E1014), isopropyl-β-d-thiogalactopyranoside (IPTG) (Sigma-Aldrich, catalog no. I6758-5G), Ni–nitrilotriacetic acid agarose (QIAGEN Ltd., catalog no. 30210), 0.25-ml ATP solution (100 mM) (Life Technologies, catalog no. R0441), QuikChange Lightning Site-Directed Mutagenesis Kit (Agilent Technologies, catalog no. 210518), cOmplete Protease Inhibitor Cocktail (Roche, catalog no. 11836145001), recombinant human NUDT16 protein (Abcam PLC., catalog no. ab103059; 100 μg), SARS-CoV-2 3CL protease (Mpro) (Bio-Techne R&D Systems, catalog no. E-720-050), Ub E1 enzyme (UBE1) (Bio-Techne R&D Systems, catalog no. E-304-050), human UbcH5a/UBE2D1 (Bio-Techne R&D Systems, catalog no. E2-616-100), Ub-biotinylated (Cambridge Bioscience Ltd., catalog no. 7551-50), recombinant human Ub (Bio-Techne R&D Systems, catalog no. U-100H-10M), recombinant human Ub mutant G76A (Bio-Techne R&D Systems, catalog no. UM-G76A-100), adenosine 5′-diphosphoribose sodium salt (Merck Life Science, catalog no. A0752-25MG), adenosine (Merck Life Science, catalog no. A9251), 2′-deoxyadenosine (Insight Biotechnology, catalog no. sc-216290), cordycepin (Cambridge Bioscience, catalog no. 14426), 2′-deoxy-ADPR (Enzo Life Sciences, catalog no. BLG-D227-01), 2′-deoxy-NAD^+^ (Enzo Life Sciences, catalog no. BLG-N065-01), NAD^+^ phosphate (Sigma-Aldrich, catalog no. 10128031001), ^32^P-NAD^+^ (Hartmann Analytic GmbH, catalog no. FP-821), ^32^P-NAD^+^ (PerkinElmer, catalog no. NEG023X250UC), and β-nicotinamide adenine dinucleotide NAD^+^ (New England Biolabs, catalog no. B9007S).

### Plasmids and mutagenesis

The genes encoding DTX2 RING-DTC (residues 390 to 622) and DTX3L RING-DTC (residues 544 to 740), with an N-terminal His_6_-tag and SARS-CoV-2 PLpro, with a C-terminal His_6_-tag were introduced into pET28a vector for *E. coli* expression. USP2 catalytic domain was transferred from pDONR221 into the pDEST17 vector using the LR Clonase II Enzyme Mix (Thermo Fisher Scientific). pET plasmids for N-terminally His_6_- and small Ub-like modifier (SUMO)-tagged UBCH5A (SUMO-UBCH5A) and N-terminally His_6_-tagged UBE1 were ordered from Addgene (no. 61081 and no. 34965, respectively), where they were deposited by C. Wolberger. Site-directed mutagenesis was performed using the QuikChange Lightning (Agilent) and confirmed by sequencing.

### Protein expression and purification

Protocols for generating UBE1 and SUMO-UBCH5A proteins were described previously (*62*, *63*). USP2 and DTX2 and DTX3L RING-DTC were expressed in *E. coli* Rosetta (DE3) cells in LB, and cultures were induced with 300 μM IPTG when the optical density at 600 nm (OD_600_) reached 0.6 to 0.8 and expressed at 18°C overnight. Harvested cells were resuspended in lysis buffer [50 mM Hepes (pH 7.5), 500 mM NaCl, and 4 mM 2-mercaptoethanol]. For expression of SARS-CoV-2 PLpro, cells were induced by addition of 500 μM IPTG and 1 mM ZnCl_2_ when OD reached 0.6 to 0.8 and further grown overnight at 18°C and harvested. The cell pellet was resuspended in lysis buffer [50 mM tris-HCl (pH 7.5), 150 mM NaCl, 10 mM imidazole, and 2 mM dithiothreitol (DTT) (pH 8.5)]. All cell suspensions were stored at −80°C until purification.

For purification, cell suspensions were thawed, supplemented with benzonase (Novagen), lysozyme, and cOmplete EDTA-free protease inhibitor cocktail (Roche) and lysed by end-over-end mixing for 1 hour at 4°C, followed by EmulsiFlex-C5 homogenizer (Avestin). Lysate was cleared by centrifugation for 60 min at 35,000*g* and incubated with Ni^2+^-agarose for 1 hour at 4°C. Resins were washed with lysis buffer supplemented with 30 mM imidazole, followed by protein elution with the same buffer containing 300 mM imidazole. All proteins were further purified by size exclusion chromatography (SEC) on a Superdex 200 column (GE Healthcare) before snap-freezing in liquid nitrogen and storing at −80°C. The final SEC buffer was 20 mM Hepes (pH 7.5), 200 mM NaCl, and 1 mM DTT (for USP2, DTX2, and DTX3L) or 20 mM tris-HCl (pH 7.5), 100 mM NaCl, 1 mM tris(2-carboxyethyl)phosphine (TCEP) (for PLpro).

Human ADPr hydrolases (PARG, ARH1, ARH2, ARH3, MACROD1, MACROD2, and TARG1), SARS-CoV-2 Macro, and PARP10CAT were produced recombinantly before in our laboratory (*40*, *53*, *64*). SARS-CoV-2 3CL protease (Mpro; E-720-050 from R&D Systems) and the following recombinant human proteins: NUDT16 (100 μg; ab103059 from Abcam), UBE1 (E-304-050 from R&D Systems), UBCH5A (E2-616-100 from R&D Systems), processed recombinant Ub (WT and G76A; U-100H-10M and UM-G76A-100, respectively, from R&D Systems), and biotinylated Ub (7551-50 from Cambridge Bioscience) were obtained from indicated commercial suppliers. All protein concentrations were determined by measuring absorption at 280 nm using NanoDrop ND-1000 (Thermo Fisher Scientific).

### NAD^+^ ubiquitylation assay

NAD^+^ ubiquitylation assays were performed at 37°C in 50 mM Hepes (pH 7.5), 50 mM NaCl, 5 mM MgCl_2_, 1 mM DTT, and 1 mM ATP containing 5 μM DTX2 RING-DTC or DTX3L RING-DTC, 0.5 μM UBE1, 2.5 μM UBCH5A, 10 μM Ub, and 50 μM NAD^+^ spiked with ^32^P-NAD^+^. Only for experiments in Fig. 1C and fig. S1 (A and B) was cold NAD^+^ omitted. After incubation at 37°C for 30 min, the reaction mixtures were resolved by SDS–polyacrylamide gel electrophoresis (SDS-PAGE). The gel was stained with Coomassie brilliant blue and dried for autoradiography.

### Ub-NAD^+^ hydrolase assay

To obtain NAD^+^-Ub, 10 μM Ub was incubated with 0.5 μM UBE1, 2.5 μM UBCH5A, and 2 μM DTX2 RING-DTC or DTX3L RING-DTC, supplemented with 1 mM ATP and 50 μM NAD^+^ spiked with ^32^P-NAD^+^ (PerkinElmer) in the reaction buffer [50 mM Hepes (pH 7.5), 50 mM NaCl, 5 mM MgCl_2_, and 1 mM DTT] at 37°C for 30 min. Then, NH_2_OH or various hydrolases (different ADPr hydrolases, SARS-CoV-2 Mpro, SARS-CoV-2 PLpro, and USP2) were added for a further 30-min treatment, while NH_2_OH and USP2 served as positive controls. All reactions were stopped by addition of SDS-PAGE loading dye and sample boiling. The samples were resolved by SDS-PAGE and analyzed by autoradiography.

For fig. S1A, reactions were supplemented with buffer, PLpro, or PLpro C111A and incubated for the indicated time before being quenched with lithium dodecyl sulfate (LDS) sample buffer (Life Technologies) and analyzed by SDS-PAGE and autoradiography. For Fig. 2B, NUDT16 and 5 mM MgCl_2_ (needed for the full activity of NUDT16) were added to reactions.

### In vitro ADP-ribosylation assay

For PARP1 and PARP1 E988Q automodification, 1 μM PARP1 or PARP1 E988Q was incubated in the reaction buffer [50 mM tris-HCl (pH 8.0), 100 mM NaCl, 2 mM MgCl_2_, 1 μM DNA duplex (5′-ATCAGATAGCATCTGTGCGGCCGCTTAGGG-3′ and 5′-CCCTAAGCGGCCGCACAGATGCTATCTGAT-3′), and 50 μM NAD^+^ spiked with ^32^P-NAD^+^], and reactions were incubated at 37°C for 30 min and then stopped by addition of the LDS sample buffer (Life Technologies) and incubation at 95°C for 5 min. Samples were then analyzed by SDS-PAGE and autoradiography.

### In vitro autoubiquitylation assay

For the in vitro autoubiquitylation assay, 5 μM DTX2 RING-DTC was incubated with 0.5 μM UBE1, 2.5 μM UBCH5A, and 10 μM Ub in 50 mM Hepes (pH 7.5), 50 mM NaCl, 5 mM MgCl_2_, 1 mM DTT, and 1 mM ATP. After incubation at 37°C for indicated time, the reaction mixtures were analyzed via SDS-PAGE and subsequent immunoblotting. In the experiment in Fig. 3H (gels shown in fig. S10B), UBCH5A was replaced with SUMO-tagged UBCH5A (WT, D87A, or D117A), labeled as “SUMO-E2.”

### Ubiquitylation of ADP-ribosylated peptide or protein

For Fig. 5C, 2 μg of H3 peptide Ac-ARTKQTARKSTGGKAPRKQLAGGK(biotin)-Am mono(ADP-ribosyl)ated (biotin-peptide-ADPr) at Ser^10^ or a control unmodified peptide with the same sequence (biotin-peptide) was mixed with 5 μM DTX2 RING-DTC, 0.5 μM UBE1, 2.5 μM UBCH5A, and 10 μM Ub in 50 mM Hepes (pH 7.5), 50 mM NaCl, 5 mM MgCl_2_, 1 mM DTT, and 1 mM ATP. After incubation for 30 min at 37°C, the reaction mixtures were resolved by SDS-PAGE, stained with Coomassie brilliant blue, and analyzed by Western blotting. For ARH3 and SARS-CoV-2 PLpro treatment, 7.5 μM ARH3 WT or ARH3 D77/78N and 5 μM PLpro WT or PLpro C111A were added to the reactions, respectively, and further incubated for 30 min at 37°C. To probe the ubiquitinated products, Western blotting using anti-Ub (sc-8017, Insight Biotechnology) and anti-biotin (ab7403, Abcam) antibodies was performed.

For the experiment in Fig. 5D, a similar procedure was followed, but a larger amount of H3 peptides (8 μg) was used and the E2 UBCH5A was replaced with a SUMO-tagged version of UBCH5A in either WT or mutant form (either at 4 μM).

For the experiment in Fig. 5E, 2.5 μM PARP10CAT was incubated at 37°C for 1 hour with or without 50 μM NAD^+^ to generate substrates for DTX2. The reaction mixture comprised 5 μM DTX2 RING-DTC, 0.5 μM UBE1, 2.5 μM WT or D117A SUMO-UBCH5A, and 10 μM Ub spiked with 1 μM biotin-Ub in 50 mM Hepes (pH 7.5), 50 mM NaCl, 5 mM MgCl_2_, 1 mM DTT, and 1 mM ATP. Following 1-hour incubation at 37°C, the reaction samples were resolved by SDS-PAGE and visualized with immunoblotting.

### Competitive MS

Combinations of NAD^+^ and ADP-ribose in different ratios (the total concentration of both was set to 1 mM) were incubated with 5 μM DTX2 RING-DTC, 0.5 μM UBE1, 2.5 μM UBCH5A, and 10 μM Ub in 50 mM Hepes (pH 7.5), 50 mM NaCl, 5 mM MgCl_2_, 1 mM DTT, and 1 mM ATP for 30 min at 37°C. The reactions were acidified by mixing in a ratio 1:9 1% trifluoroacetic acid (TFA) and were analyzed by MS.

### Effects of NAD^+^ or ADP-ribose on the inhibition of DTX2 autoubiquitylation

Increasing amounts of NAD^+^ or ADP-ribose were mixed with DTX2 reaction mixtures [5 μM DTX2 RING-DTC, 0.5 μM UBE1, 2.5 μM UBCH5A, and 10 μM Ub in 50 mM Hepes (pH 7.5), 50 mM NaCl, 5 mM MgCl_2_, and 1 mM DTT]. Ubiquitylation was triggered by adding 1 mM ATP and allowed to proceed for 30 min at 37°C. The reaction samples were resolved by SDS-PAGE and visualized with autoradiography.

### Western blotting

For Western blot analysis, samples were subjected to a standard SDS-PAGE method followed by protein transfer onto polyvinylidene difluoride membranes. Membranes were then blocked with PBST buffer [25 mM tris-HCl (pH 7.5), 150 mM NaCl, 0.5% Tween 20, and 5% nonfat dried milk] and incubated overnight with primary antibodies at 4°C, followed by 1-hour incubation with peroxidase-conjugated secondary antibodies at room temperature. For detecting biotinylated products, membranes were blocked with 3% bovine serum albumin in 25 mM tris-HCl (pH 7.5), 150 mM NaCl, and 0.5% Tween 20 and incubated with streptavidin (HRP) (ab7403).

### Semipreparative HPLC purifications

Semipreparative HPLC purifications were carried out on a LaChromElite system equipped with a Hitachi L-2130 pump, a Hitachi L-2455 diode array detector, and a Hitachi L-2200 auto sampler.

### HPLC-MS analyses

HPLC-MS analyses were carried out on an Agilent 1260 Infinity HPLC system, coupled with an Agilent 6120 mass spectrometer [electrospray ionization (ESI) + mode]. The multiply charged envelope was deconvoluted using the charge deconvolution tool in Agilent OpenLab CDS ChemStation software to obtain the average [*M*] value.

### High-resolution electrospray MS

High-resolution electrospray MS (ESI-HRMS) was performed on a maXis ultrahigh-resolution quadrupole orthogonal acceleration–time-of-flight mass spectrometer (Bruker Daltonics, Bremen, Germany).

### NMR analyses

NMR analyses were performed at 298 K, either on a Bruker Avance III HD 700-MHz NMR spectrometer equipped with a 5-mm TCI cryoprobe or a Bruker AVANCE III 600 instrument. Processing and analyses were performed with Bruker’s TopSpin 3.6 and MestreNova 12.0.4. Chemical shifts are reported in parts per million from low to high field. Coupling constants (*J*) are reported in hertz. Standard abbreviations indicating multiplicity were used as follows: s, singlet; d, doublet; dd, doublet of doublets; t, triplet; m, multiplet; and b, broad signal.

### HPLC and MS monitoring of the enzymatic reactions

Individual compounds were incubated with 5 μM DTX2 RING-DTC, 0.5 μM UBE1, 2.5 μM UBCH5A, and 10 μM Ub in 50 mM Hepes (pH 7.5), 50 mM NaCl, 5 mM MgCl_2_, 1 mM DTT, and 1 mM ATP. After incubation at 37°C for 30 min, 12-μl reactions were mixed with 2 μl of 1% TFA and subjected to HPLC-MS analysis (Aeris Widepore XB-C18 2 column; 3.6 μm, 200 Å, 150 mm by 2.1 mm, 0.5 ml/min of flow rate, and 60°C). As mobile phases, mixtures of 0.1% formic acid in H_2_O (A′) and 0.1% formic acid in MeCN (B′) were used. Gradient is as follows: 3% B′ for 1 min and then 3 to 50% B′ over 15 min.

When the different Ub-derived compounds were chromatographically separable, their relative ratio were evaluated by integration of the different peaks at 214 nm, neglecting differences in molar absorption coefficients (see fig. S2A for a representative example). When compounds were coeluted, the relative ratio was evaluated by MS by measuring peak heights on the deconvoluted spectra, neglecting differences in ionization (see fig. S2B for a representative example).

### HPLC purification and NMR analysis of Gly-Gly-ADPr

Ub (0.4 mM) and ADPr (4.44 mM) were incubated overnight at 37°C in 50 mM Hepes (pH 7.5) (8.5 ml) in the presence of 2 μM UBE1, 4 μM UBCH5A, 6 μM E3 (DTX2), 4 mM ATP, 50 mM NaCl, 5 mM MgCl_2_, and 1 mM DTT. The reaction mixture was buffer-exchanged using Zeba spin columns into 10 mM Hepes (pH 7.5) and 100 mM NaCl to get rid of the excess of ADP-ribose, ATP, and AMP (a by-product of the E1 reaction). We added 1 ml (^1^/_10th_ of the reaction mixture volume) of trypsin (1 mg/ml) (Promega; sequencing grade, V5111) and incubated the mixture for 10 min at 37°C to partially cleave GG-ADP-ribose off. The reaction was acidified by mixing it in a ratio of 1:20 with 10% TFA, and the solution was cleared by centrifugation. The mixture was purified by reversed-phase HPLC using a Gemini C18, 5 μM, 110 Å, 250-mm by 10-mm column at a flow rate of 3 ml/min, with the following gradient: 1% solvent B during 5 min and then 1 to 70% solvent B in 20 min, solvent A being 0.1% TFA in water and solvent B being 0.1% TFA in acetonitrile. Retention time was 6.51 min.

The pooled fractions were lyophilized to give 95 μg of a white solid, which was redissolved in 220 μl of DMSO-*d*6 and transferred into a 3-mm NMR tube. 1D ^1^H and 2D homonuclear ^1^H correlation spectroscopy and TOCSY (80 ms) and heteronuclear ^13^C-HSQC (natural abundance) spectra were acquired on the Bruker Avance III HD 700 spectrometer.

NMR data indicated that the purified fractions were an 85:15 mixture of two compounds, identified later to be AMP and Gly-Gly-ADPr, respectively, as evidenced from analysis of NMR and HRMS data. Traces of other minor contaminants (such as an extra Gly-Gly–containing compound; see, for example, TOCSY spectrum; fig. S8) are also observed, but the set of 2D experiments that were implemented made possible an unambiguous measurement of the chemical shifts of most ^1^H and ^13^C signals of the ADPr derivative, giving strong evidences for a Gly-Gly ester at position 3′.

Pure ADPr, AMP, and glycyl-glycine ethyl ester (Gly-Gly-OEt) hydrochloride were used as reference compounds for ^1^H and ^13^C NMR chemical shifts. Before analysis, they were dissolved in a 0.1% TFA aqueous solution (0.1 mg/ml) and then lyophilized. Spectra were recorded on the Bruker AVANCE III 600 instrument. ^13^C NMR chemical shifts were obtained from the HSQC spectra, and thus, only ribose CH and CH_2_ signals are described and compared. Full details of the analysis are provided in the Supplementary Materials.

### AlphaFold2 models

The AlphaFold2 model of the DTX2:E2 complex shown in Fig. 3C was produced using ColabFold (AlphaFold2 + MMseqs2) (*65*) with default settings (3 recycles, etc.) except for one optional feature that was included: AMBER relaxation. Sequences of the previously crystallized human DTX2 RING-DTC fragment (residues 390 to 622 of DTX2_HUMAN) and the full-length human UBCH5A (UB2D1_HUMAN) were used. The five generated models, one of which is shown in Fig. 3C, converged on an almost identical structure.

The AlphaFold2 model of the human RNF213 fragments 4545 to 4596 was created using the same settings as above. The AlphaFold2 models of human UBE2J2 and HOIL1 are shown according to the AlphaFold Protein Structure Database (alphafold.ebi.ac.uk), except for the position of His^510^ of HOIL1, which was adjusted according to the models of murine and zebrafish HOIL1 from the same database.

## Supplementary Materials

### This PDF file includes:

Figs. S1 to S11 Tables S1 to S3 NMR and HRMS characterization data References

## Notes

View/request a protocol for this paper from *Bio-protocol*.
