# Supplementary material for: DELTEX E3 ligases ubiquitylate ADP-ribosyl modification on protein substrates
Source: Sci Adv. 2022 Oct 5;8(40):eadd4253. doi: 10.1126/sciadv.add4253 (PMC7615817; doi:10.1126/sciadv.add4253)
Supplement: Supplementary file 1 [file sciadv.add4253_sm.pdf]

Science Advances
AAAS

Supplementary Materials for

DELTEX E3 ligases ubiquitylate ADP-ribosyl modification on protein substrates

Kang Zhu *et al.*

Corresponding author: Ivan Ahel, ivan.ahel@path.ox.ac.uk; Dragana Ahel, dragana.ahel@path.ox.ac.uk; Vincent Aucagne, vincent.aucagne@cnrs-orleans.fr; Marcin J. Suskiewicz, marcin.suskiewicz@cnrs-orleans.fr

Sci. Adv. **8**, eadd4253 (2022)
DOI: 10.1126/sciadv.add4253

This PDF file includes:

Figs. S1 to S11
Tables S1 to S3
NMR and HRMS characterization data
References

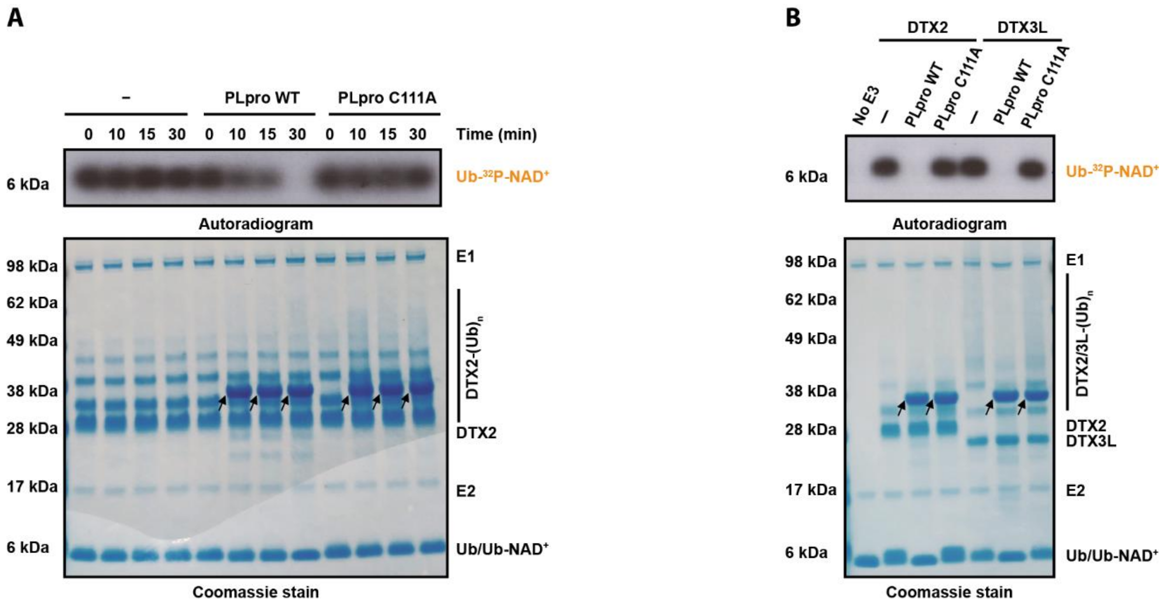

Figure S1: Ub-NAD$^+$ hydrolysis depends on the catalytic cysteine of SARS-CoV-2 PLpro

(A) SARS-CoV-2 PLpro-catalysed hydrolysis of the DTX reaction product depends on the PLpro catalytic cysteine. Ub-NAD$^+$ obtained as in **Fig. 1B** was treated with WT or catalytically dead PLpro (C111A) for indicated times. The arrows indicate PLpro.

(B) Repeat of the experiment in **A** (a final timepoint) using Ub-NAD$^+$ generated with RING DTC fragments of either DTX2 or DTX3L.

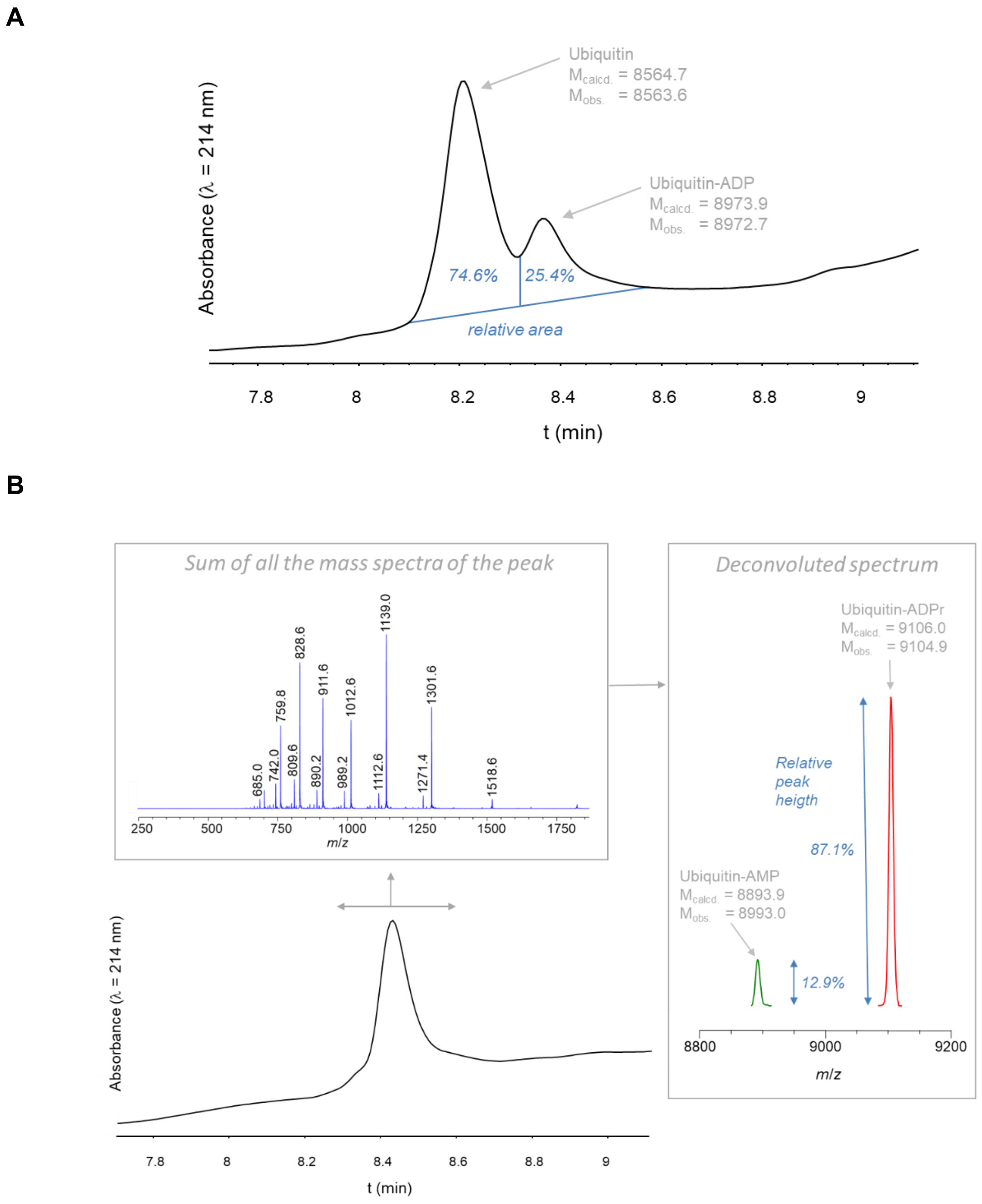

Figure S2: HPLC-MS analysis of the products of DTX-catalysed ubiquitylation.
Two representative scenarios are presented.
(A) Reaction of Ub with 4 mM ADP in the presence of DTX2 RING DTC, E1, E2 and 2 mM ATP. Two distinct peaks can be distinguished in the HPLC chromatogram, each corresponding to one predominant component identified with MS (Ub and Ub-ADP, respectively). The relative abundance of the two molecules was estimated by peak area integration.
(B) Reaction of Ub with an equimolar mixture of ADP and AMP (500 μM each) in the presence of DTX2 RING DTC, E1, E2 and 2 mM ATP. A single peak is observed in the HPLC chromatogram, but a deconvoluted MS spectrum indicates the presence of two different components (Ub-AMP and Ub-ADP-ribose). Their relative abundance was estimated based on the relative peak height in the deconvoluted spectrum.

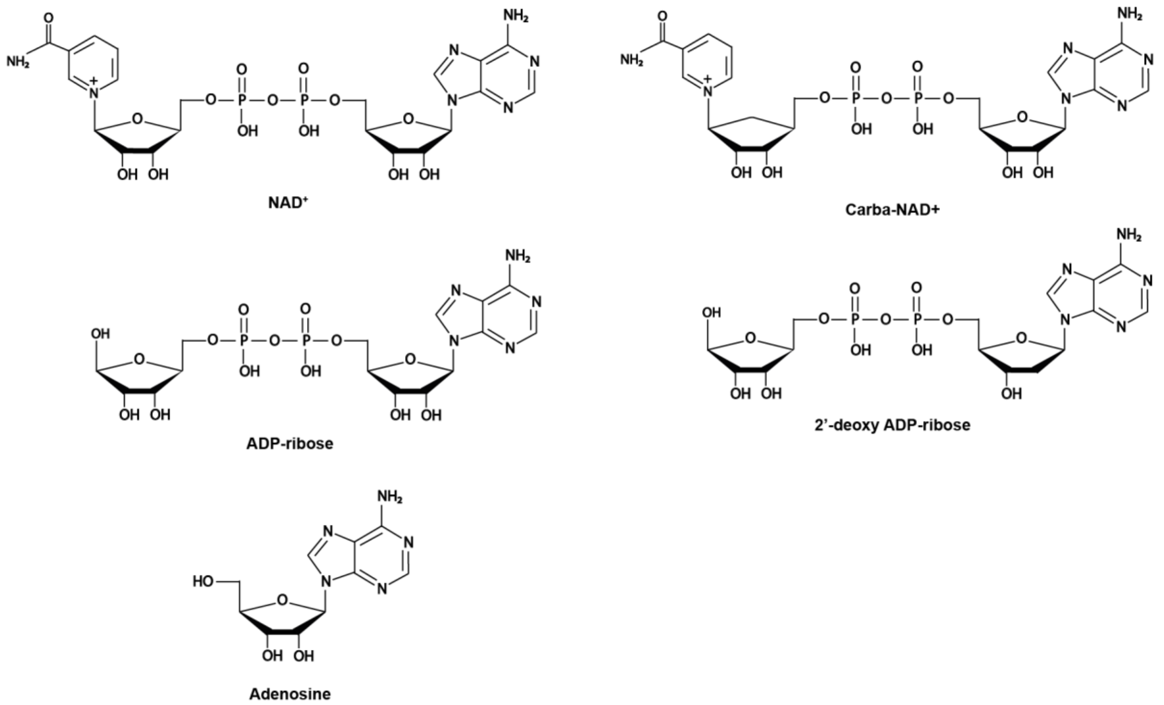

Figure S3: Chemical formulas of NAD^+, Carba-NAD^+, ADP-ribose, 2'-deoxy ADP-ribose and adenosine.

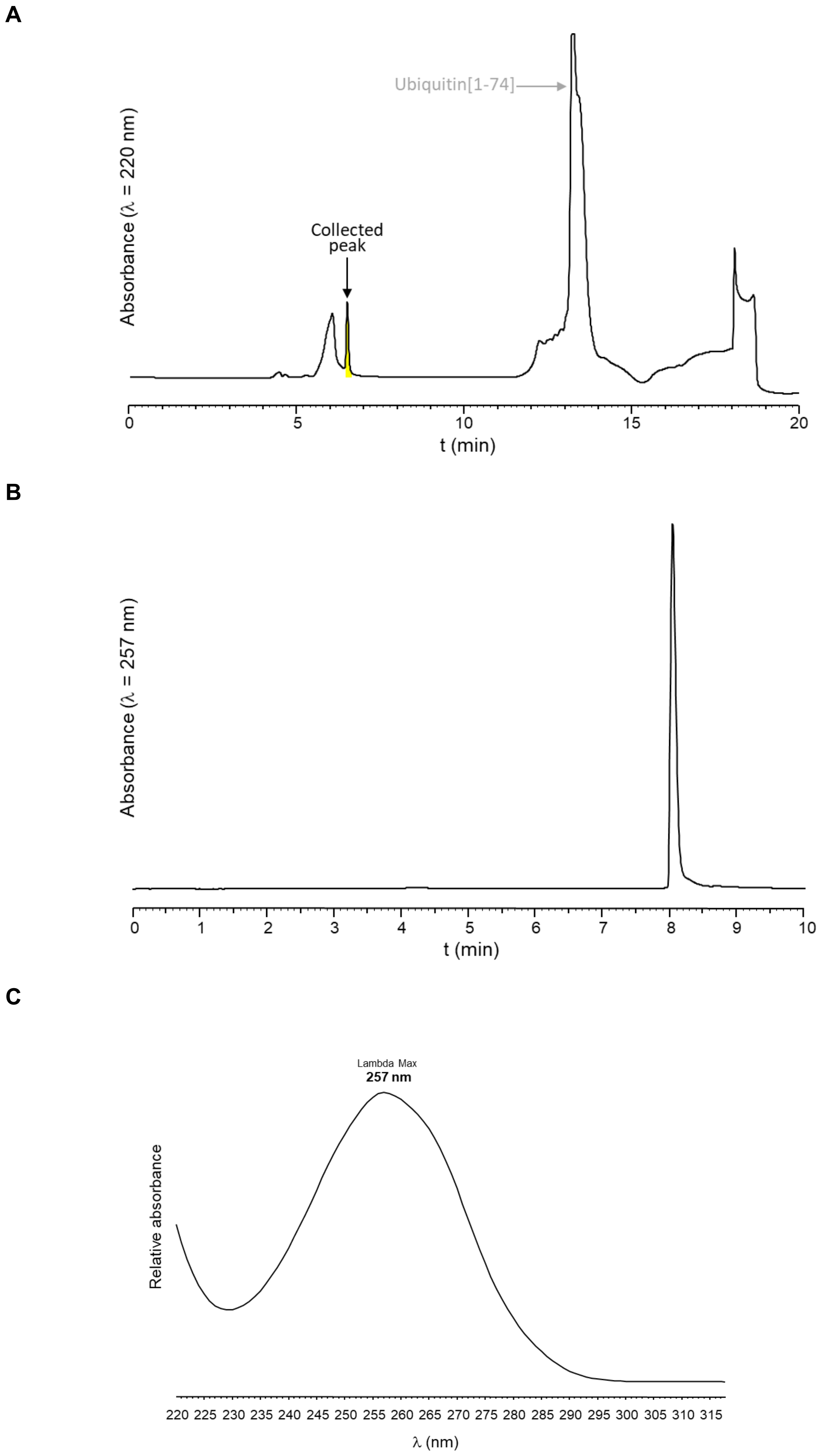
A
Ubiquitin[1-74]
Collected peak
Absorbance (λ = 220 nm)
0
5
10
15
20
t (min)
B
Absorbance (λ = 257 nm)
0
1
2
3
4
5
6
7
8
9
10
t (min)
C
Lambda Max
257 nm
Relative absorbance
220 225 230 235 240 245 250 255 260 265 270 275 280 285 290 295 300 305 310 315
λ (nm)

Figure S4: HPLC purification of Gly-Gly-ADP-ribose.

(A) Representative HPLC chromatogram obtained when separating the crude reaction mixture following limited digestion with trypsin.

(B) Chromatogram of the purified fraction containing Gly-Gly-ADP-ribose and AMP. A Gemini C18 column with a gradient 0 to 10% B in 10 minutes was used.

(C) UV spectrum of Gly-Gly-ADPr extracted from the diode array detector signal of the HPLC peak shown in B.

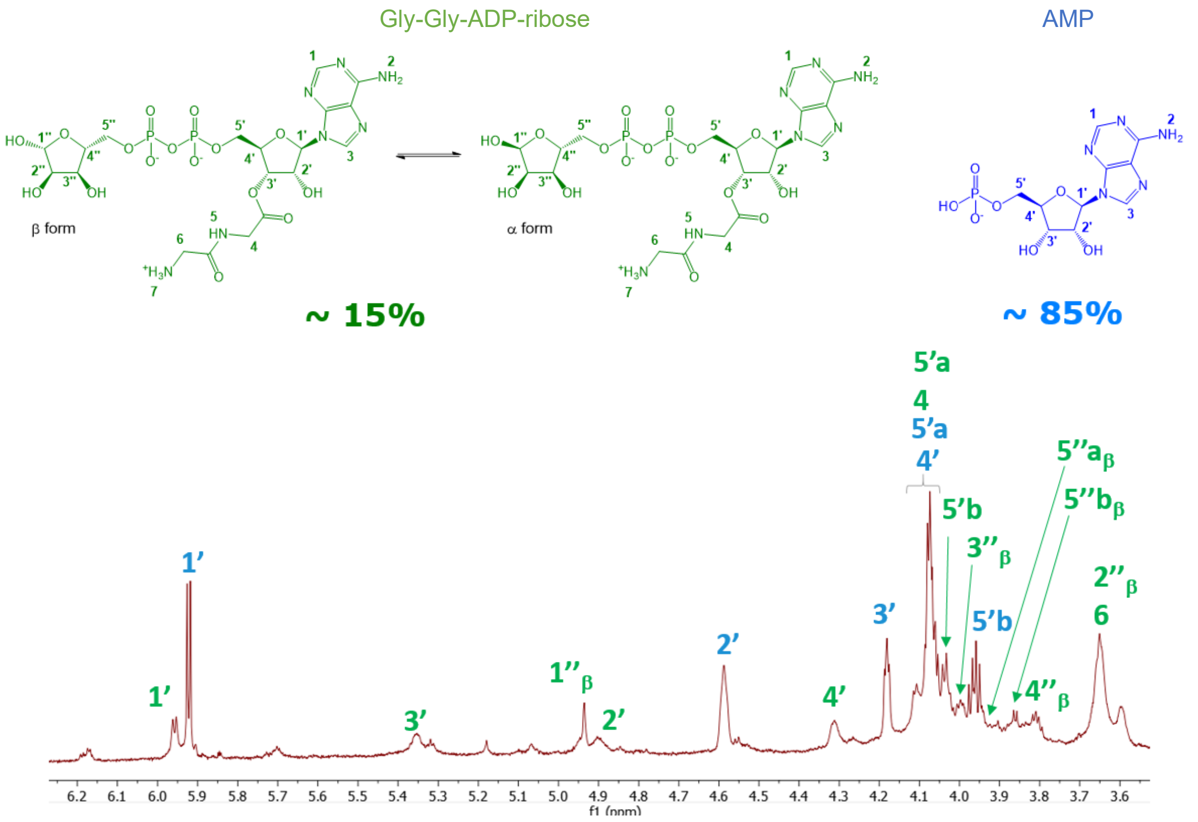

Figure S5: ^{1}H spectrum of the isolated Gly-Gly-ADP-ribose:AMP mixture.

A zoomed-in fragment of the spectrum is presented. Positions within ADP-ribose (β or α anomers, in case of differences between the two) and AMP to which the peaks can be mapped are indicated. The Y axis corresponds to intensity.

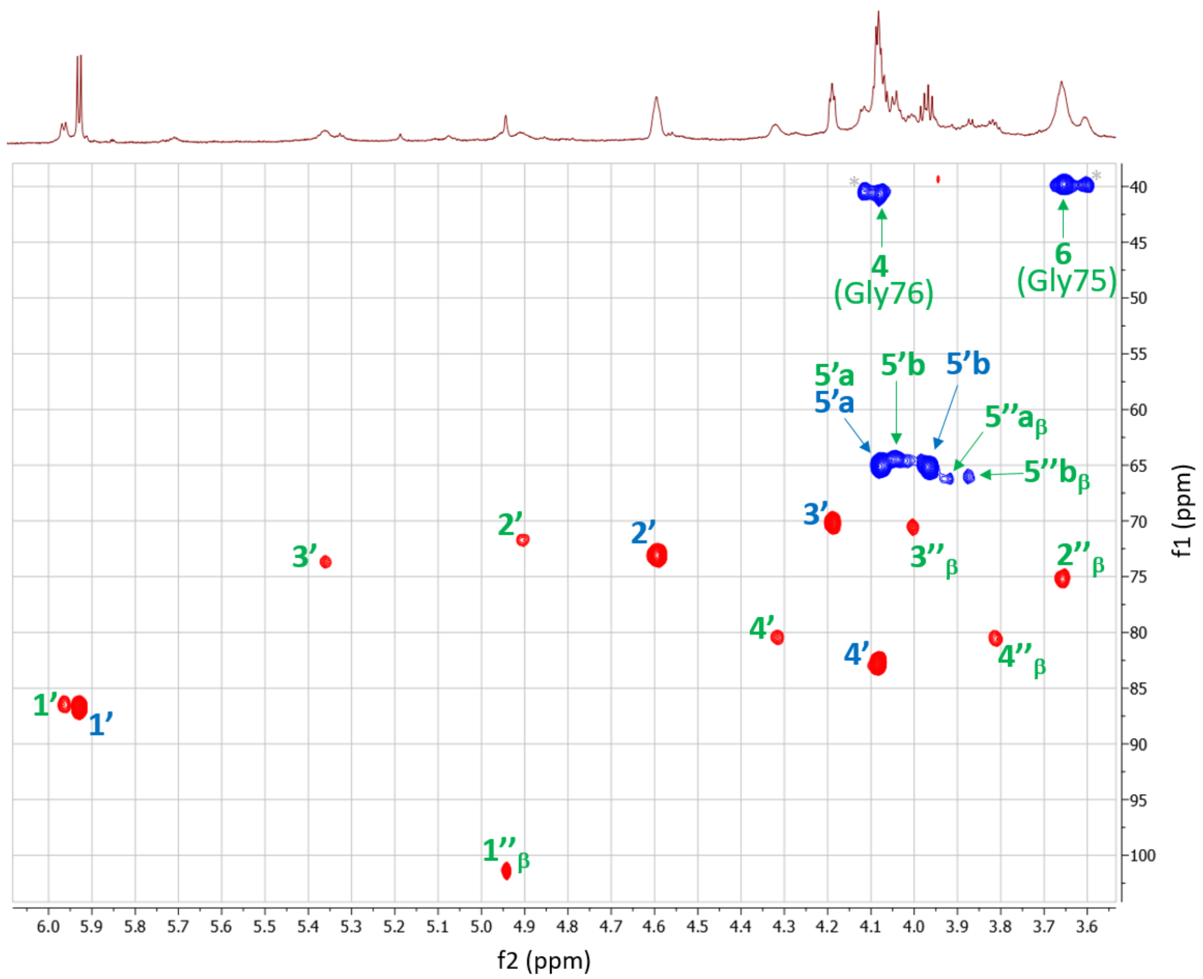

Figure S6: Phase-edited ^{1}H-^{13}C HSQC of the isolated Gly-Gly-ADP-ribose:AMP mixture. Cross-peaks corresponding to CH and CH_2 appear in red and blue, respectively. Blue cross-peaks near the Gly-Gly CH_2 signals of Gly-Gly-ADP-ribose (H4 and H6), and highlighted with a grey asterisk, likely come from a contaminant also containing a Gly-Gly moiety.

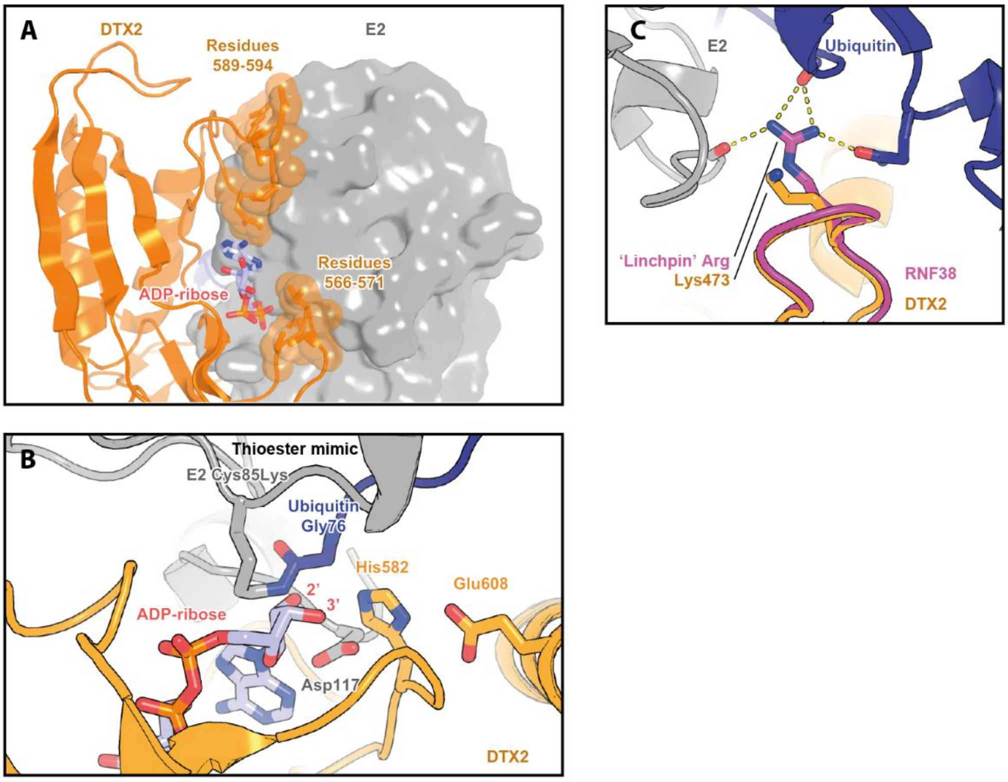

Figure S7: Details of the structural model of the DTX2:E2~Ubiquitin complex.

Zoomed-in views of the model of the DTX2:E2~Ubiquitin complex from **Fig. 3A**, created by alignment of PDB entries 6Y3J and 4V3L on the RING domain.

A) The loop regions of DTX2 (shown as semi-transparent spheres and sticks) might clash with E2 (shown in surface representation) unless they adopt a different conformation or DTX2 and E2 move further apart from each other.

B) The active site of the DTX2:E2~Ub complex. The Cys85Lys mutation was introduced in the E2 in PDB 4V3L in order to create a stable mimic of the E2~Ub thioester.

C) Lys473 of DTX2 is located in the "linchpin" position of the RING domain, where an arginine residue is canonically present. As shown for the linchpin arginine of RNF38, this residue can stabilise E2 and Ub relative to each other by simultaneously interacting with both these proteins via hydrogen bonds (shown as yellow dashes). The Lys473 conformation is shown as in the DTX2 RING DTC structure in which E2~Ub is not present (PDB 6Y3J). Upon binding to E2~Ub, Lys473 likely adopts a different rotamer and partially recapitulates the interactions that are made by the linchpin arginine residue.

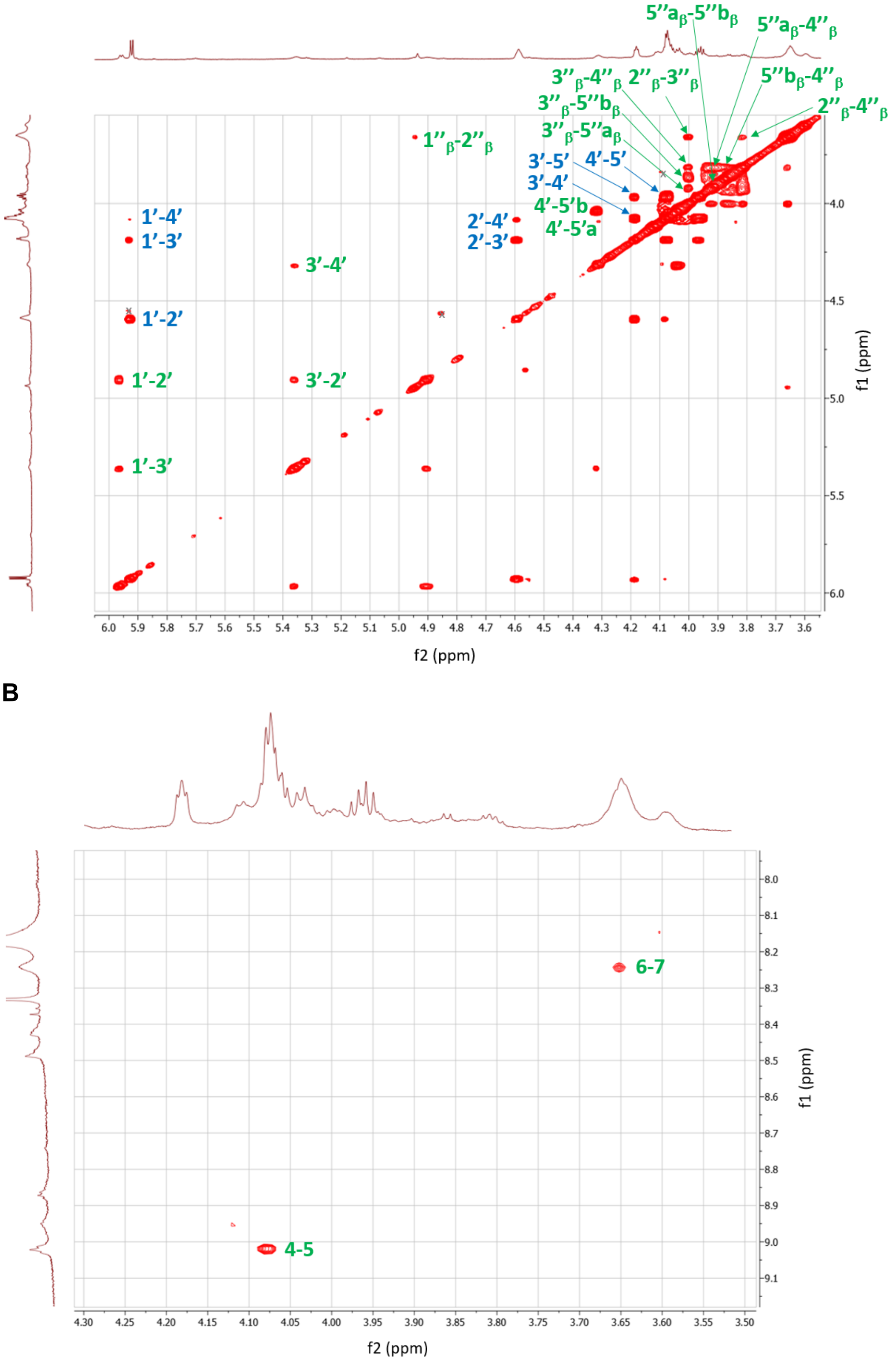

Figure S8: ^{1}H TOCSY of the isolated Gly-Gly-ADP-ribose:AMP mixture.

(A) Full 2D spectrum.

(B) Zoom on the Gly signals.

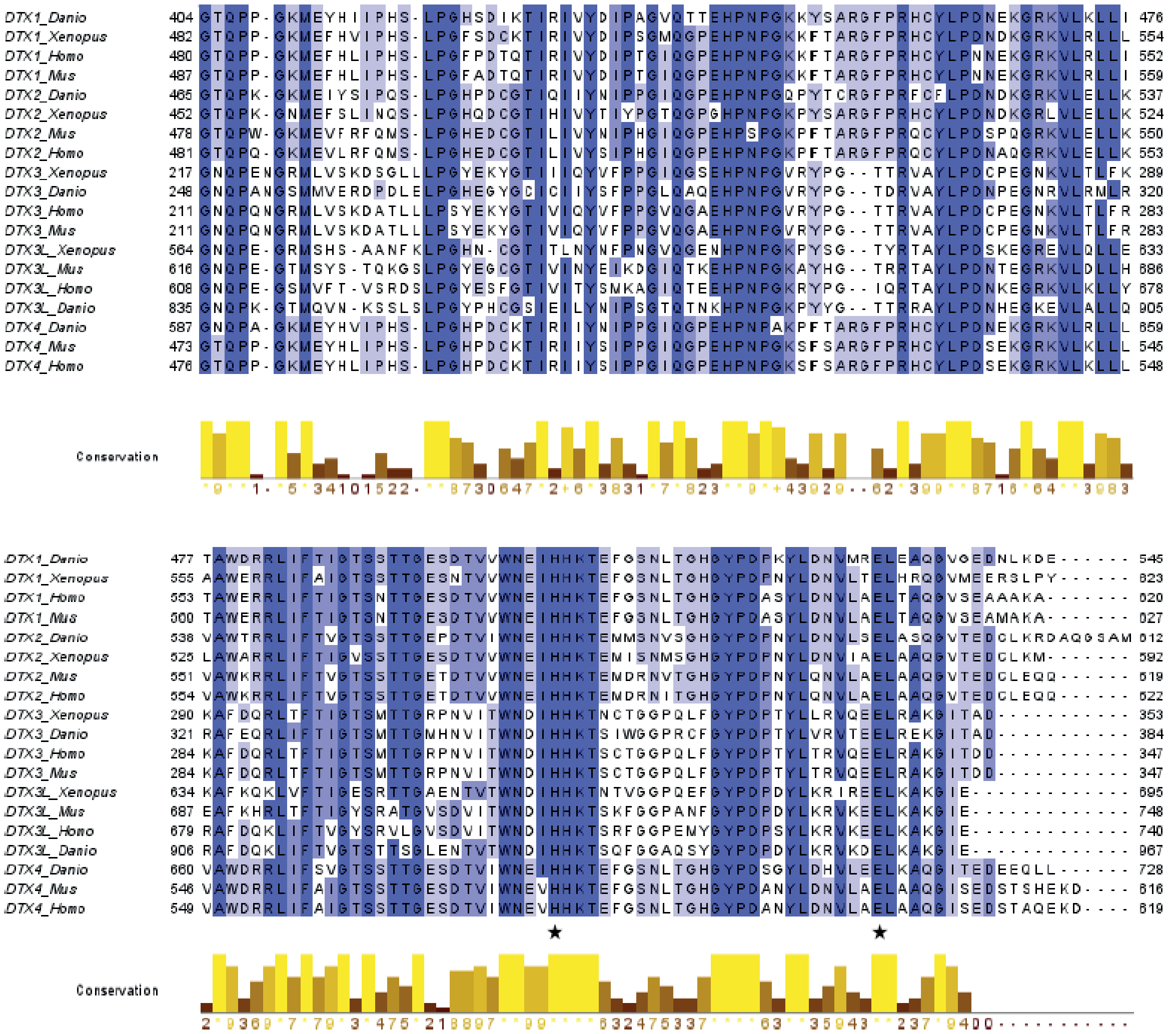

Figure S9: Multiple sequence alignment of DTX DTC domains.

Sequences of DTC domains from different DTX paralogues (DTX1, 2, 3, 3L, 4) from four model organisms (*Danio* = *Danio rerio*; *Xenopus* = *Xenopus laevis*; *Mus* = *Mus musculus*; *Homo* = *Homo sapiens*) were aligned in Jalview, version 2.8.1, using default settings. Residues are coloured with shades of blue according to their conservation and a conservation score is presented underneath. The strictly conserved catalytic residues E608 and H582 (human DTX2 numbering) are marked with stars.

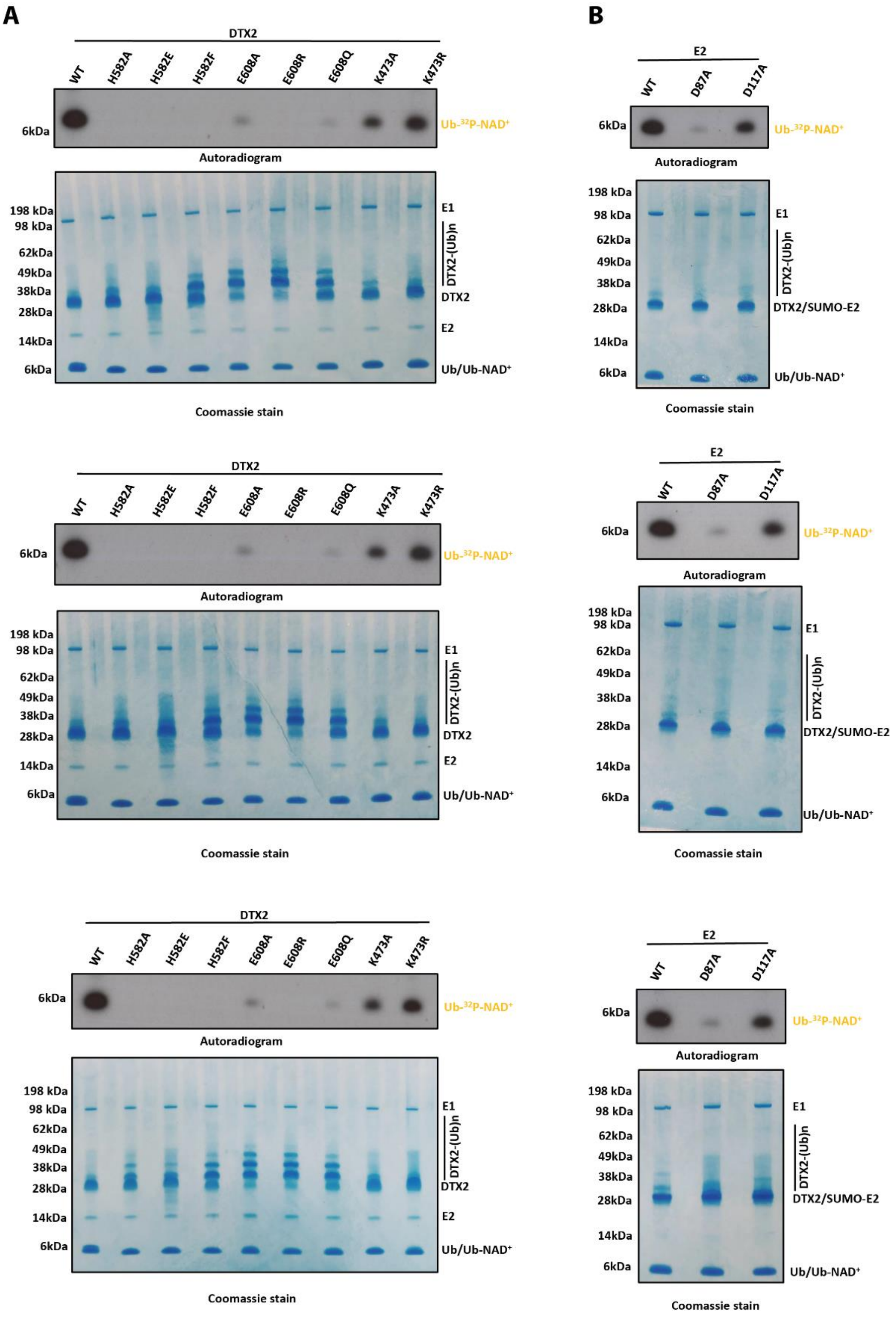

Figure S10: Mutational analysis of the active-site residues of DTX2 and E2

The complete data (three repeats of the experiment performed with the same protein preparations) to accompany **Fig. 3D** and **H**, where a representative autoradiogram is shown for each experiment alongside the quantification of results from these repeats. We used the NAD$^+$ ubiquitylation assay introduced in **Fig. 1B**. Results were visualized with autoradiography and Coomassie staining.

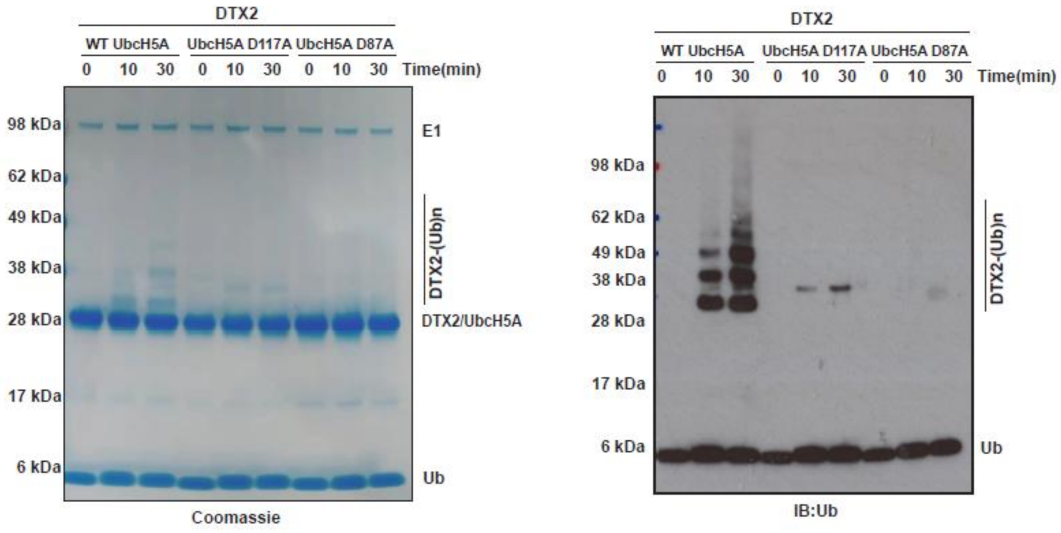

Figure S11: Autoubiquitylation of DTX2 RING DTC depends on Asp117 and Asp87 of the E2 UBCH5A.

DTX2 RING DTC was incubated with E1, Ub and ATP for the indicated times and autoubiquitylation visualised with both a Coomassie stain and anti-Ub antibody. Dependence on both Asp117 and Asp87 suggests that DTX2 automodifies on lysine residues.

δ ^{1}H (ppm)

		Reference compounds			Major cpnd (AMP)	Δppm / AMP	Minor cpnd (Gly-Gly-ADPr)	Δppm / ADPr	Δppm / Gly-Gly-OEt
		AMP	ADPr[a]	Gly-Gly-OEt					
H1		8.33	8.39	-	*NI* [b]	-	*NI* [b]	-	-
H3		8.49	8.61	-	*NI* [b]	-	*NI* [b]	-	-
H1'		5.96	5.95	-	5.92	-0.04	5.97	0.02	-
H2'		4.57	4.55	-	4.59	0.02	4.90	0.35	-
H3'		4.19	4.23	-	4.17	-0.02	5.36	1.13	-
H4'		4.10	4.14	-	4.08	-0.02	4.32	0.18	-
H5'a		4.08	4.16	-	4.07	-0.01	4.11	-0.05	-
H5'b		3.98	4.11	-	3.97	-0.01	4.05	-0.06	-
H1''	β	-	4.94	-	-	-	4.94	0	-
	α	-	5.08	-	-	-	5.08	0	-
H2''	β	-	3.65	-	-	-	3.67	0.02	-
	α	-	3.81	-	-	-	3.82	0.01	-
H3''	β	-	3.95	-	-	-	4.00	0.05	-
	α	-	3.95	-	-	-	3.94	-0.01	-
H4''	β	-	3.83	-	-	-	3.82	-0.01	-
	α	-	3.83	-	-	-	*NI* [b]	-	-
H5''a	β	-	4.05	-	-	-	3.93	-0.12	-
	α	-	3.96	-	-	-	*NI* [b]	-	-
H5''b	β	-	3.90	-	-	-	3.87	-0.03	-
	α	-	3.90	-	-	-	*NI* [b]	-	-
H4		-	-	3.90	-	-	4.08	-	0.18
H5		-	-	9.05	-	-	9.02	-	-0.03
H6		-	-	3.58	-	-	3.64	-	0.06
H7		-	-	8.35	-	-	8.24	-	-0.11

a: 77:23 mixture of β and α anomers
b: these peak were Not Identified unambiguously

Table S1: Summary of ^{1}H chemical shifts (DMSO-*d*6) for both major (AMP, 85%) and minor compound (Gly-Gly-ADPr, 15%) of the isolated mixture.

δ ^{13}C (ppm)

	Reference compounds			Major cpnd (AMP)	Δppm / AMP	Minor cpnd (Gly-Gly-ADPr)	Δppm / ADPr	Δppm / Gly-Gly-OEt
	AMP	ADPr	Gly-Gly-OEt					
C1'	87.7	87.8	-	87.5	-0.2	87.2	-0.6	-
C2'	74.0	74.3	-	73.8	-0.2	72.5	-1.8	-
C3'	70.8	70.6	-	70.8	0	74.4	3.8	-
C4'	83.7	84.0	-	83.5	-0.2	81.1	-2.9	-
C5'	65.8	66.0	-	65.8	0	65.2	-0.8	-
C1'' β	-	102.2	-	-	-	102.2	0	-
C2'' β	-	75.7	-	-	-	75.9	0.2	-
C3'' β	-	71.3	-	-	-	71.3	0	-
C4'' β	-	80.8	-	-	-	81.3	0.5	-
C5'' β	-	68.6	-	-	-	66.9	-1.7	-
C4	-	-	41.1	-	-	41.2	-	0.1
C6	-	-	40.4	-	-	40.5	-	0.1

Table S2: Summary of the comparison of ^{13}C chemical shifts (DMSO-*d*6) for both major (AMP, 85%) and minor compound (Gly-Gly-ADPr, 15%) of the isolated mixture.

	Ethanol CH_3-CH_2-OH	Ethyl acetate CH_3-CH_2-$OCOCH_3$	Δ (ppm) ester *vs* alcohol
CH_2	56.07	59.74	+ 3.67
CH_3	18.51	14.4	- 4.11

Table S3: Comparison of ^{13}C chemical shifts of ethyl alcohol and an ester derivative (DMSO-*d*6) showing similar tendencies as observed in Gly-Gly-ADP-ribose *versus* ADP-ribose: upshift of the carbon in α to the oxygen, and downshift of the β.

The data are from ref. (*66*).

NMR and HRMS characterisation data

Note that only non-ambiguously identified NMR signals from the AMP/GG-ADPr mixture are reported. Coupling constants could be clearly measured for only some of the peaks, due to the overlap of the signals in the mixture. To facilitate comparisons, multiplets are not reported as a range of chemical shifts as is customary, but rather as a more precise and indicative median chemical shift, whose measurement was made possible from the ^{1}H-^{13}C HSQC spectrum.

Gly-Gly-ADPr

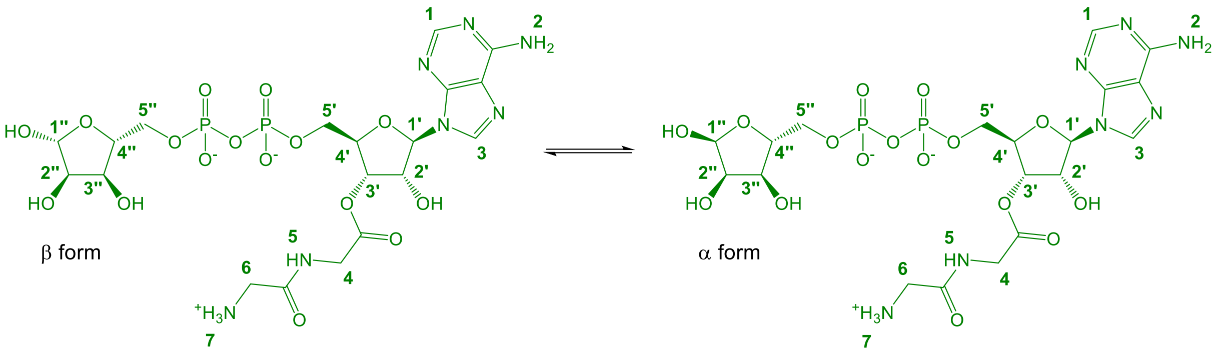

ESI- HRMS (*m/z*): $[M-H]^-$ calcd. for $C_{19}H_{28}N_7O_{16}P_2$: 672.1073, found 672.1076 (monoisotopic)

ESI- MS (*m/z*): $[M-H]^-$ calcd. for $C_{19}H_{28}N_7O_{16}P_2$: 672.1, found 672.0 (monoisotopic)

ESI+ MS (*m/z*): $[M+H]^+$ calcd. for $C_{19}H_{30}N_7O_{16}P_2$: 674.1, found 674.0 (monoisotopic)

As expected, Gly-Gly-ADP-ribose was observed as an ~8:2 mixture of β and α anomers (integration of $H_{1''}$ signals), which are described separately for ease of comparison with ADP-ribose. As one could expect, we were able to characterise unambiguously only a few signals from the α anomer, most of them not being distinct from those of the β form, as reported later in the manuscript for ADP-ribose.

<u>β anomer</u>:

^{1}H NMR (700 MHz, DMSO-*d*6): δ 9.02 (bt, *J* = 5.8 Hz, 1H, H_5), 8.24 (bs, 3H, H_7), 5.97 (d, *J* = 5.7 Hz, 1H, $H_{1'}$), 5.36 (m, 1H, $H_{3'}$), 4.94 (m, 1H, $H_{1''}$), 4.90 (m, 2H, $H_{2'}$), 4.32 (m, 1H, $H_{4'}$), 4.11 (m, 1H, $H_{5'a}$), 4.08 (m, 2H, H_4), 4.05 (m, 1H, $H_{5'b}$), 4.00 (m, 1H, $H_{3'}$), 3.93 (m, 1H, $H_{5''a}$), 3.87 (m, 1H, $H_{5''b}$), 3.82 (m, 1H, $H_{4''}$), 3.67 (m, 1H, $H_{2''}$), 3.65 (m, 2H, H_6).

^{13}C NMR (175 MHz, DMSO-*d*6): δ 102.2 ($C_{1''}$), 87.2 ($C_{1'}$), 81.3 ($C_{4''}$), 81.1 ($C_{4'}$), 75.9 ($C_{2''}$), 74.4 ($C_{3'}$), 72.5 ($C_{2'}$), 71.3 ($C_{3''}$), 66.9 ($C_{5''}$), 65.2 ($C_{5'}$), 41.2 (C_6), 40.5(C_4).

α anomer:

Only the peaks that were distinct from those of the β anomer, and that were unambiguously assigned from the TOCSY spectrum are reported.

^{1}H NMR (600 MHz, DMSO-*d*6): δ 5.08 (m, 1H, $H_{1''}$), 3.94 (m, 1H, $H_{3''}$), 3.82 (m, 1H, $H_{2''}$).

AMP

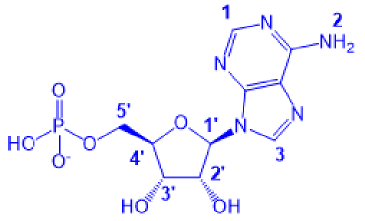

Data obtained from the mixture with Gly-Gly-ADP-ribose:

ESI- HRMS (*m*/*z*): $[M-H]^-$ calcd. for $C_{10}H_{13}N_5O_7P$: 346.0558, found 346.0560

^{1}H NMR (700 MHz, DMSO-*d*6): δ 5.92 (d, *J* = 5.7 Hz, 1H, $H_{1'}$), 4.59 (bt, *J* = 5.2 Hz, 1H, $H_{2'}$), 4.17 (dd, *J* = 4.9, 3.9 Hz, 1H, $H_{3'}$), 4.08 (m, 1H, $H_{4'}$), 4.07 (m, 1H, $H_{5'a}$), 3.97 (m, 1H, $H_{5'b}$).

^{13}C NMR (175 MHz, DMSO-*d*6): δ 87.5 ($C_{1'}$), 83.5 ($C_{4'}$), 73.8 ($C_{2'}$), 70.8 ($C_{3'}$), 65.9 ($C_{5'}$).

Data obtained from a pure commercial compound:

^{1}H NMR (600 MHz, DMSO-*d*6): δ 8.49 (s, 1H, H_3), 8.33 (s, 1H, H_1), 5.96 (d, *J* = 5.6 Hz, 1H, $H_{1'}$), 4.57 (t, *J* = 5.3 Hz, 1H, $H_{2'}$), 4.19 (dd, *J* = 5.0, 3.6 Hz, 1H, $H_{3'}$), 4.10 (m, 1H, $H_{4'}$), 4.08 (m, 1H, $H_{5'a}$), 3.98 (ddd, *J* = 11.1, 6.4, 4.6 Hz, 1H, $H_{5'b}$).

^{13}C NMR (150 MHz, DMSO-*d*6): δ 87.7 ($C_{1'}$), 83.7 ($C_{4'}$), 74.0 ($C_{2'}$), 70.8 ($C_{3'}$), 65.8 ($C_{5'}$).

ADP-ribose (data obtained from a pure commercial compound)

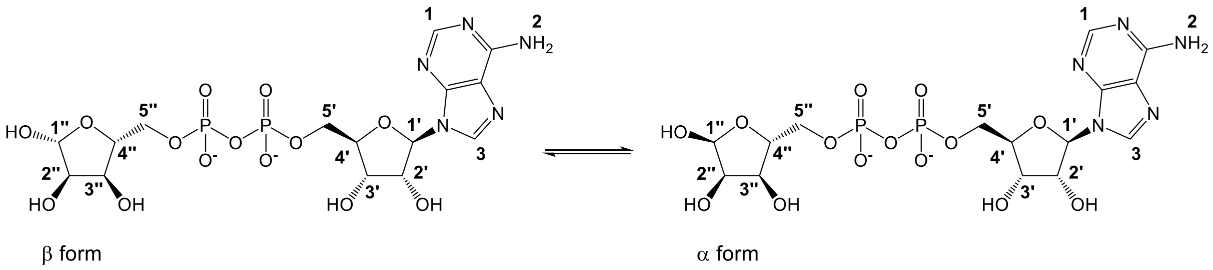

ADP-ribose was observed as a 74:26 mixture of β and α anomers, which are described separately for ease of comparison with Gly-Gly-ADP-ribose, for which we were able to characterise unambiguously only the major β anomer.

β anomer:

^{1}H NMR (600 MHz, DMSO-*d6*): δ 8.61 (s, 1H, H_3), 8.39 (s, 1H, H_1), 5.95 (d, *J* = 5.4 Hz, 1H, $H_{1'}$), 4.94 (d, *J* = 1.5 Hz, 1H, $H_{1''}$), 4.55 (dd, *J* = 5.2, 5.2 Hz, 2H, $H_{2'}$), 4.23 (m, 1H, $H_{3'}$), 4.16 (m, 1H, $H_{5'a}$), 4.14 (m, 1H, $H_{4'}$), 4.11 (m, 1H, $H_{5'b}$), 4.05 (ddd, *J* = 10.2, 6.1, 3.6 Hz, 1H, $H_{5''a}$), 3.95 (dd, *J* = 6.7, 4.7 Hz, 1H, $H_{3''}$), 3.90 (ddd, *J* = 10.8, 6.4, 6.4 Hz, 1H, $H_{5''b}$), 3.83 (dt, *J* = 6.7, 3.4 Hz, 1H, $H_{4''}$), 3.65 (dd, *J* = 4.7, 1.5 Hz, 1H, $H_{2''}$).

^{13}C NMR (150 MHz, DMSO-*d6*): δ 102.2 ($C_{1''}$), 87.8 ($C_{1'}$), 84.0 ($C_{4'}$), 80.8 ($C_{4''}$), 75.7 ($C_{2''}$), 74.3 ($C_{2'}$), 71.3 ($C_{3''}$), 70.6 ($C_{3'}$), 68.6 ($C_{5''}$), 66.0 ($C_{5'}$).

α anomer:

Signals from the adenosine moiety (carbons and protons 1, 3 and 1'-5') were undistinguishable from those of the β anomer, and their description is not repeated.

^{1}H NMR (600 MHz, DMSO-*d6*): δ 5.08 (d, *J* = 4.0 Hz, 1H, $H_{1''}$), 3.96 (m, 1H, $H_{5''a}$), 3.95 (m, 1H, $H_{3'}$), 3.90 (m, 1H, $H_{5''b}$), 3.83 (m, 1H, $H_{4''}$), 3.81 (m, 1H, $H_{2''}$).

^{13}C NMR (150 MHz, DMSO-*d6*): δ 96.5 ($C_{1''}$), 80.8 ($C_{4''}$), 71.3 ($C_{3''}$), 70.9 ($C_{2''}$), 66.6 ($C_{5''}$).

Gly-Gly-OEt.HCl (data obtained from the pure commercial compound)

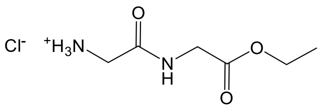

^{1}H NMR (600 MHz, DMSO-*d6*): δ 9.05 (t, *J* = 5.9 Hz, 2H, NH), 8.35 (s, 3H, NH_3^+), 4.09 (q, *J* = 7.1 Hz, 2H, CH_2-O), 3.90 (d, *J* = 5.9 Hz, 2H, CH_2-NH), 3.58 (bs, 1H, CH2-NH_3^+), 1.18 (t, *J* = 7.1 Hz, 3H, CH_3).

^{13}C NMR (150 MHz, DMSO-*d6*): δ 169.8 (COO), 167.0 (CONH), 61.1 (CH_2-O), 40.4 (CH_2-NH_3^+), 41.1 (CH_2-NH), 14.5 (CH_3).

REFERENCES AND NOTES

1. A. Hershko, A. Ciechanover, The ubiquitin system. *Annu. Rev. Biochem.* **67**, 425–479 (1998).

2. D. Komander, M. Rape, The ubiquitin code. *Annu. Rev. Biochem.* **81**, 203–229 (2012).

3. J. N. Pruneda, K. E. Stoll, L. J. Bolton, P. S. Brzovic, R. E. Klevit, Ubiquitin in motion: Structural studies of the ubiquitin-conjugating enzyme∼ubiquitin conjugate. *Biochemistry* **50**, 1624–1633 (2011).

4. N. Zheng, N. Shabek, Ubiquitin ligases: Structure, function, and regulation. *Annu. Rev. Biochem.* **86**, 129–157 (2017).

5. E. Branigan, J. Carlos Penedo, R. T. Hay, Ubiquitin transfer by a RING E3 ligase occurs from a closed E2~ubiquitin conformation. *Nat. Commun.* **11**, 2846 (2020).

6. A. Plechanovova, E. G. Jaffray, S. A. McMahon, K. A. Johnson, I. Navratilova, J. H. Naismith, R. T. Hay, Mechanism of ubiquitylation by dimeric RING ligase RNF4. *Nat. Struct. Mol. Biol.* **18**, 1052–1059 (2011).

7. J. N. Pruneda, P. J. Littlefield, S. E. Soss, K. A. Nordquist, W. J. Chazin, P. S. Brzovic, R. E. Klevit, Structure of an E3: E2∼Ub complex reveals an allosteric mechanism shared among RING/U-box ligases. *Mol. Cell* **47**, 933–942 (2012).

8. J. Ahel, A. Fletcher, D. B. Grabarczyk, E. Roitinger, L. Deszcz, A. Lehner, S. Virdee, T. Clausen, E3 ubiquitin ligase RNF213 employs a non-canonical zinc finger active site and is allosterically regulated by ATP. bioRxiv 2021.05.10.443411 [**Preprint**]. 10 May 2021. https://doi.org/10.1101/2021.05.10.443411.

9. J. Ahel, A. Lehner, A. Vogel, A. Schleiffer, A. Meinhart, D. Haselbach, T. Clausen, Moyamoya disease factor RNF213 is a giant E3 ligase with a dynein-like core and a distinct ubiquitin-transfer mechanism. *eLife* **9**, e56185 (2020).

10. K. C. Pao, N. T. Wood, A. Knebel, K. Rafie, M. Stanley, P. D. Mabbitt, R. Sundaramoorthy, K. Hofmann, D. M. F. van Aalten, S. Virdee, Activity-based E3 ligase profiling uncovers an E3 ligase with esterification activity. *Nature* **556**, 381–385 (2018).

11. I. R. Kelsall, J. Zhang, A. Knebel, J. S. C. Arthur, P. Cohen, The E3 ligase HOIL-1 catalyses ester bond formation between ubiquitin and components of the Myddosome in mammalian cells. *Proc. Natl. Acad. Sci. U.S.A.* **116**, 13293–13298 (2019).

12. I. R. Kelsall, E. H. McCrory, Y. Xu, C. L. Scudamore, S. K. Nanda, P. Mancebo-Gamella, N. T. Wood, A. Knebel, S. J. Matthews, P. Cohen, HOIL-1 ubiquitin ligase activity targets unbranched glucosaccharides and is required to prevent polyglucosan accumulation. *EMBO J.* **41**, e109700 (2022).

13. E. G. Otten, E. Werner, A. Crespillo-Casado, K. B. Boyle, V. Dharamdasani, C. Pathe, B. Santhanam, F. Randow, Ubiquitylation of lipopolysaccharide by RNF213 during bacterial infection. *Nature* **594**, 111–116 (2021).

14. V. De Cesare, D. Carbajo Lopez, P. D. Mabbitt, A. J. Fletcher, M. Soetens, O. Antico, N. T. Wood, S. Virdee, Deubiquitinating enzyme amino acid profiling reveals a class of ubiquitin esterases. *Proc. Natl. Acad. Sci. U.S.A.* **118**, e2006947118 (2021).

15. X. Wang, R. A. Herr, M. Rabelink, R. C. Hoeben, E. J. H. J. Wiertz, T. H. Hansen, Ube2j2 ubiquitinates hydroxylated amino acids on ER-associated degradation substrates. *J. Cell Biol.* **187**, 655–668 (2009).

16. M. Schuller, I. Ahel, Beyond protein modification: The rise of non-canonical ADP-ribosylation. *Biochem. J.* **479**, 463–477 (2022).

17. L. Song, Z. Q. Luo, Post-translational regulation of ubiquitin signaling. *J. Cell Biol.* **218**, 1776–1786 (2019).

18. E. Barkauskaite, G. Jankevicius, I. Ahel, Structures and mechanisms of enzymes employed in the synthesis and degradation of PARP-dependent protein ADP-ribosylation. *Mol. Cell* **58**, 935–946 (2015).

19. E. Prokhorova, T. Agnew, A. R. Wondisford, M. Tellier, N. Kaminski, D. Beijer, J. Holder, J. Groslambert, M. J. Suskiewicz, K. Zhu, J. M. Reber, S. C. Krassnig, L. Palazzo, S. Murphy, M. L. Nielsen, A. Mangerich, D. Ahel, J. Baets, R. J. O'Sullivan, I. Ahel, Unrestrained poly-ADP-ribosylation provides insights into chromatin regulation and human disease. *Mol. Cell* **81**, 2640–2655.e8 (2021).

20. S. Bhogaraju, S. Kalayil, Y. Liu, F. Bonn, T. Colby, I. Matic, I. Dikic, Phosphoribosylation of ubiquitin promotes serine ubiquitination and impairs conventional ubiquitination. *Cell* **167**, 1636–1649.e13 (2016).

21. J. Qiu, M. J. Sheedlo, K. Yu, Y. Tan, E. S. Nakayasu, C. Das, X. Liu, Z. Q. Luo, Ubiquitination independent of E1 and E2 enzymes by bacterial effectors. *Nature* **533**, 120–124 (2016).

22. F. Yan, C. Huang, X. Wang, J. Tan, S. Cheng, M. Wan, Z. Wang, S. Wang, S. Luo, A. Li, X. Guo, M. Feng, X. Liu, Y. Zhu, Y. Zhou, Threonine ADP-ribosylation of ubiquitin by a bacterial effector family blocks host ubiquitination. *Mol. Cell* **78**, 641–652.e9 (2020).

23. C. A. Vivelo, V. Ayyappan, A. K. L. Leung, Poly(ADP-ribose)-dependent ubiquitination and its clinical implications. *Biochem. Pharmacol.* **167**, 3–12 (2019).

24. P. A. DaRosa, Z. Wang, X. Jiang, J. N. Pruneda, F. Cong, R. E. Klevit, W. Xu, Allosteric activation of the RNF146 ubiquitin ligase by a poly(ADP-ribosyl)ation signal. *Nature* **517**, 223–226 (2015).

25. H. C. Kang, Y.-I. Lee, J.-H. Shin, S. A. Andrabi, Z. Chi, J.-P. Gagné, Y. Lee, H. S. Ko, B. D. Lee, G. G. Poirier, V. L. Dawson, T. M. Dawson, Iduna is a poly(ADP-ribose) (PAR)-dependent E3 ubiquitin ligase that regulates DNA damage. *Proc. Natl. Acad. Sci. U.S.A.* **108**, 14103–14108 (2011).

26. Y. Zhang, S. Liu, C. Mickanin, Y. Feng, O. Charlat, G. A. Michaud, M. Schirle, X. Shi, M. Hild, A. Bauer, V. E. Myer, P. M. Finan, J. A. Porter, S.-M. A. Huang, F. Cong, RNF146 is a poly(ADP-ribose)-directed E3 ligase that regulates axin degradation and Wnt signalling. *Nat. Cell Biol.* **13**, 623–629 (2011).

27. L. Wang, X. Sun, J. He, Z. Liu, Functions and molecular mechanisms of Deltex family ubiquitin E3 ligases in development and disease. *Front. Cell Dev. Biol.* **9**, 706997 (2021).

28. M. E. Zweifel, D. J. Leahy, D. Barrick, Structure and Notch receptor binding of the tandem WWE domain of Deltex. *Structure* **13**, 1599–1611 (2005).

29. Y. Ashok, C. Vela-Rodriguez, C. Yang, H. I. Alanen, F. Liu, B. M. Paschal, L. Lehtio, Reconstitution of the DTX3L-PARP9 complex reveals determinants for high-affinity heterodimerization and multimeric assembly. *Biochem. J.* **479**, 289–304 (2022).

30. K. Takeyama, R. C. T. Aguiar, L. Gu, C. He, G. J. Freeman, J. L. Kutok, J. C. Aster, M. A. Shipp, The BAL-binding protein BBAP and related Deltex family members exhibit ubiquitin-protein isopeptide ligase activity. *J. Biol. Chem.* **278**, 21930–21937 (2003).

31. S. F. Ahmed, L. Buetow, M. Gabrielsen, S. Lilla, C. Chatrin, G. J. Sibbet, S. Zanivan, D. T. Huang, DELTEX2 C-terminal domain recognizes and recruits ADP-ribosylated proteins for ubiquitination. *Sci. Adv.* **6**, eabc0629 (2020).

32. C. Chatrin, M. Gabrielsen, L. Buetow, M. A. Nakasone, S. F. Ahmed, D. Sumpton, G. J. Sibbet, B. O. Smith, D. T. Huang, Structural insights into ADP-ribosylation of ubiquitin by Deltex family E3 ubiquitin ligases. *Sci. Adv.* **6**, eabc0418 (2020).

33. C.-S. Yang, K. Jividen, A. Spencer, N. Dworak, L. Ni, L. T. Oostdyk, M. Chatterjee, B. Kuśmider, B. Reon, M. Parlak, V. Gorbunova, T. Abbas, E. Jeffery, N. E. Sherman, B. M. Paschal, Ubiquitin modification by the E3 ligase/ADP-ribosyltransferase Dtx3L/Parp9. *Mol. Cell* **66**, 503–516.e5 (2017).

34. L. C. Russo, R. Tomasin, I. A. Matos, A. C. Manucci, S. T. Sowa, K. Dale, K. W. Caldecott, L. Lehtio, D. Schechtman, F. C. Meotti, A. Bruni-Cardoso, N. C. Hoch, The SARS-CoV-2 Nsp3 macrodomain reverses PARP9/DTX3L-dependent ADP-ribosylation induced by interferon signaling. *J. Biol. Chem.* **297**, 101041 (2021).

35. Y. Zhang, D. Mao, W. T. Roswit, X. Jin, A. C. Patel, D. A. Patel, E. Agapov, Z. Wang, R. M. Tidwell, J. J. Atkinson, G. Huang, R. McCarthy, J. Yu, N. E. Yun, S. Paessler, T. G. Lawson, N. S. Omattage, T. J. Brett, M. J. Holtzman, PARP9-DTX3L ubiquitin ligase targets host histone H2BJ and viral 3C protease to enhance interferon signaling and control viral infection. *Nat. Immunol.* **16**, 1215–1227 (2015).

36. T. Klemm, G. Ebert, D. J. Calleja, C. C. Allison, L. W. Richardson, J. P. Bernardini, B. G. Lu, N. W. Kuchel, C. Grohmann, Y. Shibata, Z. Y. Gan, J. P. Cooney, M. Doerflinger, A. E. Au, T. R. Blackmore, G. J. van der Heden van Noort, P. P. Geurink, H. Ovaa, J. Newman, A. Riboldi-Tunnicliffe, P. E. Czabotar, J. P. Mitchell, R. Feltham, B. C. Lechtenberg, K. N. Lowes, G. Dewson, M. Pellegrini, G. Lessene, D. Komander, Mechanism and inhibition of the papain-like protease, PLpro, of SARS-CoV-2. *EMBO J* **39**, e106275 (2020).

37. D. Shin, R. Mukherjee, D. Grewe, D. Bojkova, K. Baek, A. Bhattacharya, L. Schulz, M. Widera, A. R. Mehdipour, G. Tascher, P. P. Geurink, A. Wilhelm, G. J. van der Heden van Noort, H. Ovaa, S. Muller, K. P. Knobeloch, K. Rajalingam, B. A. Schulman, J. Cinatl, G. Hummer, S. Ciesek, I. Dikic, Papain-like protease regulates SARS-CoV-2 viral spread and innate immunity. *Nature* **587**, 657–662 (2020).

38. J. G. M. Rack, L. Palazzo, I. Ahel, (ADP-ribosyl)hydrolases: Structure, function, and biology. *Genes Dev.* **34**, 263–284 (2020).

39. Y. M. O. Alhammad, M. M. Kashipathy, A. Roy, J.-P. Gagné, P. McDonald, P. Gao, L. Nonfoux, K. P. Battaile, D. K. Johnson, E. D. Holmstrom, G. G. Poirier, S. Lovell, A. R. Fehr, The SARS-CoV-2 conserved macrodomain is a mono-ADP-ribosylhydrolase. *J. Virol.* **95**, e01969-20 (2021).

40. J. G. M. Rack, V. Zorzini, Z. Zhu, M. Schuller, D. Ahel, I. Ahel, Viral macrodomains: A structural and evolutionary assessment of the pharmacological potential. *Open Biol.* **10**, 200237 (2020).

41. B. G. Szczepankiewicz, H. Dai, K. J. Koppetsch, D. Qian, F. Jiang, C. Mao, R. B. Perni, Synthesis of carba-NAD and the structures of its ternary complexes with SIRT3 and SIRT5. *J. Org. Chem.* **77**, 7319–7329 (2012).

42. L. Palazzo, C. M. Daniels, J. E. Nettleship, N. Rahman, R. L. McPherson, S.-E. Ong, K. Kato, O. Nureki, A. K. L. Leung, I. Ahel, ENPP 1 processes protein ADP-ribosylation in vitro. *FEBS J.* **283**, 3371–3388 (2016).

43. L. Palazzo, B. Thomas, A.-S. Jemth, T. Colby, O. Leidecker, K. L. H. Feijs, R. Zaja, O. Loseva, J. C. Puigvert, I. Matic, T. Helleday, I. Ahel, Processing of protein ADP-ribosylation by Nudix hydrolases. *Biochem. J.* **468**, 293–301 (2015).

44. Y. Zhang, J. Wang, M. Ding, Y. Yu, Site-specific characterization of the Asp- and Glu-ADP-ribosylated proteome. *Nat. Methods* **10**, 981–984 (2013).

45. K. D. Wilkinson, T. K. Audhya, Stimulation of ATP-dependent proteolysis requires ubiquitin with the COOH-terminal sequence Arg-Gly-Gly. *J. Biol. Chem.* **256**, 9235–9241 (1981).

46. L. Buetow, M. Gabrielsen, N. G. Anthony, H. Dou, A. Patel, H. Aitkenhead, G. J. Sibbet, B. O. Smith, D. T. Huang, Activation of a primed RING E3-E2-ubiquitin complex by non-covalent ubiquitin. *Mol. Cell* **58**, 297–310 (2015).

47. C. Lips, T. Ritterhoff, A. Weber, M. K. Janowska, M. Mustroph, T. Sommer, R. E. Klevit, Who with whom: Functional coordination of E2 enzymes by RING E3 ligases during poly-ubiquitylation. *EMBO J.* **39**, e104863 (2020).

48. P. D. Mabbitt, A. Loreto, M. A. Dery, A. J. Fletcher, M. Stanley, K. C. Pao, N. T. Wood, M. P. Coleman, S. Virdee, Structural basis for RING-Cys-Relay E3 ligase activity and its role in axon integrity. *Nat. Chem. Biol.* **16**, 1227–1236 (2020).

49. A. Plechanovová, E. G. Jaffray, M. H. Tatham, J. H. Naismith, R. T. Hay, Structure of a RING E3 ligase and ubiquitin-loaded E2 primed for catalysis. *Nature* **489**, 115–120 (2012).

50. A. A. Yunus, C. D. Lima, Lysine activation and functional analysis of E2-mediated conjugation in the SUMO pathway. *Nat. Struct. Mol. Biol.* **13**, 491–499 (2006).

51. J. J. Bonfiglio, P. Fontana, Q. Zhang, T. Colby, I. Gibbs-Seymour, I. Atanassov, E. Bartlett, R. Zaja, I. Ahel, I. Matic, Serine ADP-ribosylation depends on HPF1. *Mol. Cell* **65**, 932–940.e6 (2017).

52. J. J. Bonfiglio, O. Leidecker, H. Dauben, E. J. Longarini, T. Colby, P. San Segundo-Acosta, K. A. Perez, I. Matic, An HPF1/PARP1-based chemical biology strategy for exploring ADP-ribosylation. *Cell* **183**, 1086–1102.e23 (2020).

53. P. Fontana, J. J. Bonfiglio, L. Palazzo, E. Bartlett, I. Matic, I. Ahel, Serine ADP-ribosylation reversal by the hydrolase ARH3. *eLife* **6**, e28533 (2017).

54. J. G. M. Rack, Q. Liu, V. Zorzini, J. Voorneveld, A. Ariza, K. H. Ebrahimi, J. M. Reber, S. C. Krassnig, D. Ahel, G. A. van der Marel, A. Mangerich, J. S. O. McCullagh, D. V. Filippov, I. Ahel, Mechanistic insights into the three steps of poly(ADP-ribosylation) reversal. *Nat. Commun.* **12**, 4581 (2021).

55. H. Kleine, E. Poreba, K. Lesniewicz, P. O. Hassa, M. O. Hottiger, D. W. Litchfield, B. H. Shilton, B. Luscher, Substrate-assisted catalysis by PARP10 limits its activity to mono-ADP-ribosylation. *Mol. Cell* **32**, 57–69 (2008).

56. S. Vyas, I. Matic, L. Uchima, J. Rood, R. Zaja, R. T. Hay, I. Ahel, P. Chang, Family-wide analysis of poly(ADP-ribose) polymerase activity. *Nat. Commun.* **5**, 4426 (2014).

57. L. Buetow, D. T. Huang, Structural insights into the catalysis and regulation of E3 ubiquitin ligases. *Nat. Rev. Mol. Cell Biol.* **17**, 626–642 (2016).

58. R. J. Deshaies, C. A. P. Joazeiro, RING domain E3 ubiquitin ligases. *Annu. Rev. Biochem.* **78**, 399–434 (2009).

59. L. Hedstrom, Serine protease mechanism and specificity. *Chem. Rev.* **102**, 4501–4524 (2002).

60. M. J. Suskiewicz, F. Zobel, T. E. H. Ogden, P. Fontana, A. Ariza, J. C. Yang, K. Zhu, L. Bracken, W. J. Hawthorne, D. Ahel, D. Neuhaus, I. Ahel, HPF1 completes the PARP active site for DNA damage-induced ADP-ribosylation. *Nature* **579**, 598–602 (2020).

61. H. Dou, L. Buetow, G. J. Sibbet, K. Cameron, D. T. Huang, Essentiality of a non-RING element in priming donor ubiquitin for catalysis by a monomeric E3. *Nat. Struct. Mol. Biol.* **20**, 982–986 (2013).

62. C. E. Berndsen, C. Wolberger, A spectrophotometric assay for conjugation of ubiquitin and ubiquitin-like proteins. *Anal. Biochem.* **418**, 102–110 (2011).

63. R. Wiener, A. T. DiBello, P. M. Lombardi, C. M. Guzzo, X. Zhang, M. J. Matunis, C. Wolberger, E2 ubiquitin-conjugating enzymes regulate the deubiquitinating activity of OTUB1. *Nat. Struct. Mol. Biol.* **20**, 1033–1039 (2013).

64. D. Munnur, E. Bartlett, P. Mikolcevic, I. T. Kirby, J. G. M. Rack, A. Mikoc, M. S. Cohen, I. Ahel, Reversible ADP-ribosylation of RNA. *Nucleic Acids Res.* **47**, 5658–5669 (2019).

65. M. Mirdita, K. Schutze, Y. Moriwaki, L. Heo, S. Ovchinnikov, M. Steinegger, ColabFold: Making protein folding accessible to all. *Nat. Methods* **19**, 679–682 (2022).

66. H. E. Gottlieb, V. Kotlyar, A. Nudelman, NMR chemical shifts of common laboratory solvents as trace impurities. *J. Org. Chem.* **62**, 7512–7515 (1997)
